# Author Correction: The α-globin super-enhancer acts in an orientation-dependent manner

**DOI:** 10.1038/s41467-025-58071-3

**Published:** 2025-03-19

**Authors:** Mira T. Kassouf, Helena S. Francis, Matthew Gosden, Maria C. Suciu, Damien J. Downes, Caroline Harrold, Martin Larke, Marieke Oudelaar, Lucy Cornell, Joseph Blayney, Jelena Telenius, Barbara Xella, Yuki Shen, Nikolaos Sousos, Jacqueline A. Sharpe, Jacqueline Sloane-Stanley, Andrew J. H. Smith, Christian Babbs, Jim R. Hughes, Douglas R. Higgs

**Affiliations:** 1 Gene Regulation Laboratory, MRC Weatherall Institute of Molecular Medicine, John Radcliffe Hospital, OX3 9DS Oxford, UK; 2 MRC Molecular Haematology Unit, MRC Weatherall Institute of Molecular Medicine, John Radcliffe Hospital, OX3 9DS Oxford, UK; 3 Max Planck Institute for Multidisciplinary Sciences, 37077 Gottingen, Germany; 4 Institute for Regeneration and Repair, MRC Centre for Regenerative Medicine, University of Edinburgh, Edinburgh, Scotland EH16 4UU UK; 5 Chinese Academy of Medical Sciences Oxford Institute, University of Oxford, OX3 7BN Oxford, UK

**Keywords:** Transcriptional regulatory elements, Gene expression, Molecular engineering

Correction to: *Nature Communications* 10.1038/s41467-025-56380-1, published online 25 January 2025

In this article the affiliation ‘Chinese Academy of Medical Sciences Oxford Institute, University of Oxford, Oxford OX3 7BN, UK’ for Douglas R. Higgs was missing. In addition, the funding from ‘the Chinese Academy of Medical Sciences (CAMS) Innovation Fund for Medical Science (CIFMS), China (grant number: 2018-I2M-2-002)’ was omitted. The original article has been corrected.

