# Supplementary material for: The α-globin super-enhancer acts in an orientation-dependent manner
Source: Nat Commun. 2025 Jan 25;16:1033. doi: 10.1038/s41467-025-56380-1 (PMC11762767; doi:10.1038/s41467-025-56380-1)
Supplement: Supplementary file 1 — Supplementary Information [file 41467_2025_56380_MOESM1_ESM.pdf]

The α-globin super-enhancer acts in an orientation-dependent manner

Content

Supplementary Figures

Supplementary Fig. 1 The well-characterised α-globin locus, a model to study a super-enhancer functional polarity

Supplementary Fig. 2 SE^{INV} allele integrity confirmed by optical mapping and homozygous mice are viable with a phenotype reminiscent of stress erythropoiesis

Supplementary Fig. 3 Gene Expression is perturbed at the α-globin locus in the SE^{INV} E10.5 primitive erythroid cells

Supplementary Fig. 4 Comparison of NG-Capture C profiles captured from promoters and CTCF sites shows changes in interactions between WT and SE^{INV} primary definitive erythroid cells across the α-globin locus

Supplementary Fig. 5 NG-Capture C profiles show redirected interactions from all the promoters present across the α-globin locus when comparing WT and SE^{INV} primary definitive erythroid cells

Supplementary Fig. 6 NG-Capture C profiles show undisturbed interactions from all the promoters captured within 5.6Mb flanking the α-globin locus when comparing WT and SE^{INV} primary erythroid cells

Supplementary Fig. 7 Deletion of the 5' α-globin boundary element (HS3839 CTCF) in SE^{INV} mESC model does not rescue the SE^{INV} phenotype in *in vitro* derived erythroid cells

Supplementary Fig. 8 NG-Capture C profiles captured from all the promoters present within the α-globin sub-TAD show changes in interactions when comparing α-globin 5' boundary (HS3839) knock out (ΔCTCF) and HS3839 knock out in the SE inversion model (SE^{INV}-ΔCTCF) in EB-derived erythroid cells

Supplementary Fig. 9 Expression analysis and Rad21 ChIP quantitation at the α-globin locus in erythroid cells derived from WT, SE^{INV}, SE^{INV}-ΔCTCF and SE^{INV}-ΔCTCF-ΔMpg mESC models

Supplementary Fig. 10 PolII and Med1 ChIP at the α-globin locus in erythroid cells derived from WT and SE^{INV}-ΔCTCF-ΔMpg mESC models show a distribution pattern that mirrors the SE sequence and functional orientation

Supplementary Figure 11. Overview of the α-globin super-enhancer inversion genome editing strategy

Supplementary Figure 12. Southern blot data showing heterozygous (WT/ SE^{INV}) and homozygous SE^{INV} (SE^{INV} / SE^{INV}) mESCs clones before and after *Hprt* minigene excision

Supplementary Tables

Supplementary Table 1. Sequences for Southern Blot probes and Sanger Sequencing primers used for screening the SE^{INV} models

Supplementary Table 2. gRNAs used for CRISPR-Cas9 genome editing

Supplementary Table 3. Primer pairs used for PCR screening of mESC targeted clones

Supplementary Table 4. Flow cytometry and magnetic column purification antibodies

Supplementary Table 5. Antibodies used for ChIP-seq

Supplementary Table 6. Primer pairs used for expression analysis by RT-PCR

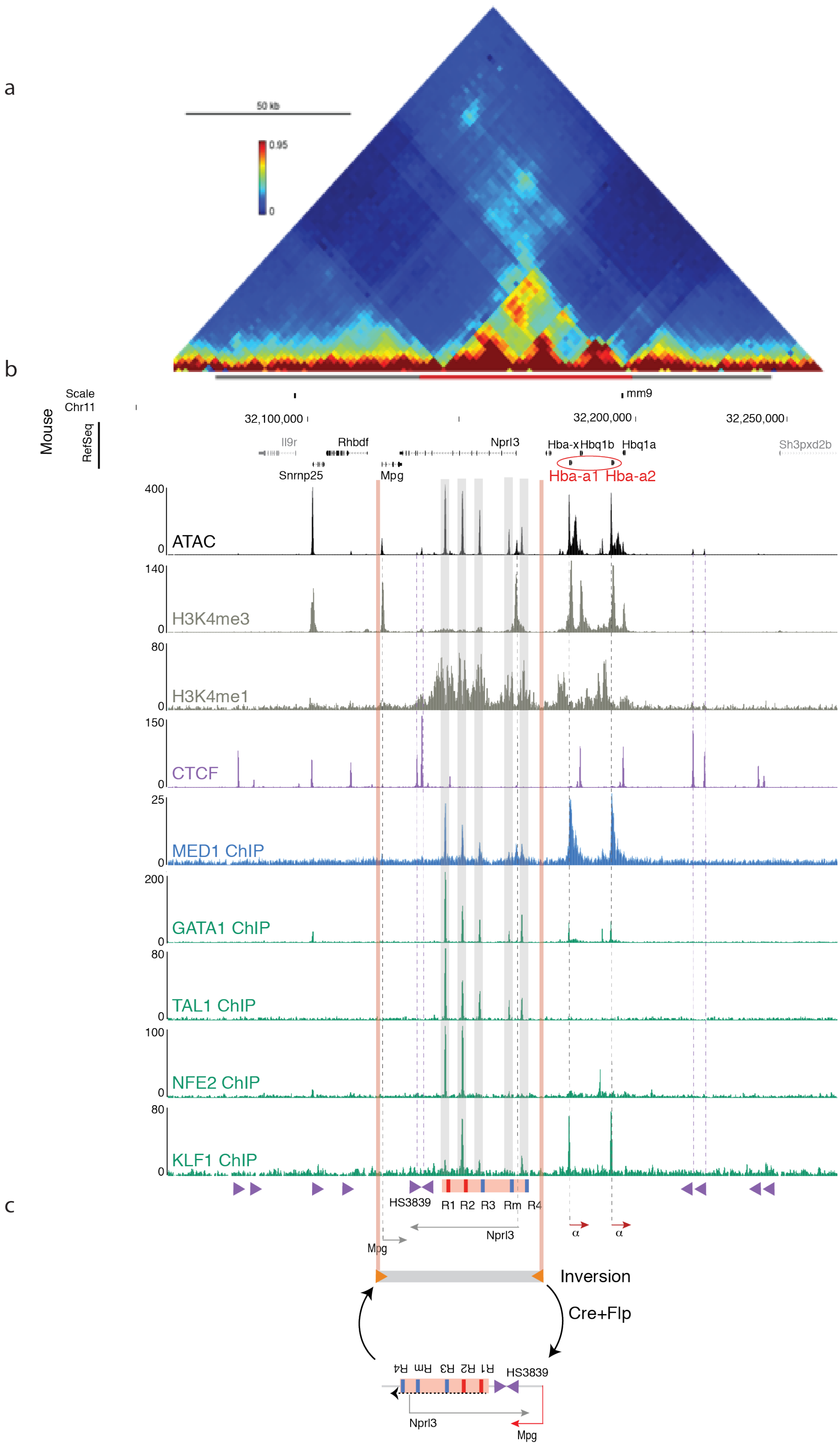

Supplementary Fig. 1

Supplementary Fig. 1 The well-characterised α-globin locus, a model to study a super-enhancer functional polarity. a Chromatin conformation capture (3C, Tiled-C Capture) contact matrix covering 200 kb spanning the mouse α-globin cluster (mm9 coordinates chr11:32,060,000-32,260,000) with the higher intensity red colours reflecting the higher frequency of contact between the α-globin major cis-regulatory TAD (chr11:32,080,000-32,245,000) and sub-TAD (chr11:32,136,000-32,202,000), respectively. **b** Accessible chromatin (ATAC-seq); histone modifications (H3K4me3 and H3K4me1); occupancy of specialized and erythroid transcription factors (CTCF, MED1, GATA1, TAL1, NFE2 and KLF1). The α-globin genes are marked in red and a red circle and the enhancers are highlighted in grey shaded vertical bars. **c** A schematic representation of the main regulatory elements spanning the α-globin locus, marked by dashed grey and purple vertical lines and indicating the adult α-globin genes (α) and flanking genes (*Nprl3, Mpg*) in red and grey arrows pointing in the direction of their expression and the CTCF-binding sites and their corresponding orientation in purple arrows, with the α-globin tested 5' boundary elements labelled (HS3839). Orange box represents the SE and the vertical red bars mark the two enhancers (R1, R2) and blue bars the facilitators (R3, Rm, R4). Orange shaded vertical bars mark the limits of the inversion encompassing the SE as well as the *Nprl3* and *Mpg* genes, an interval that was flanked by convergent heterotypic LoxP sites (grey bar flanked by orange convergent arrows) and was flipped and selection cassette excised upon sequential expression of Cre-recombinase (Cre) and Flp-recombinase (Flp) in mESCs, as shown in the schematic.

a

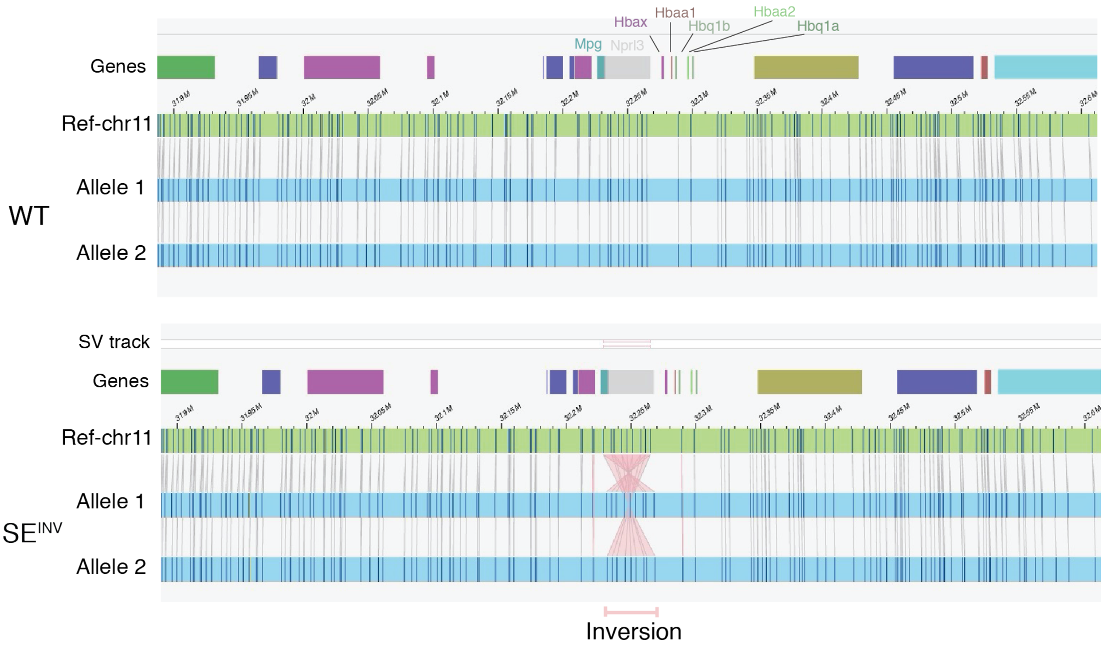

b

HetxHet	WT	Het	Hom
Expected	19.5	39	19.5
Obtained	22	38	18
**HetxHom**			
Expected	N/A	15.5	15.5
Obtained	N/A	19	12
**HomxHom**			
Obtained	N/A	N/A	46
Litter Size	N/A	N/A	~7

c

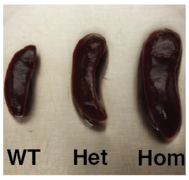

d

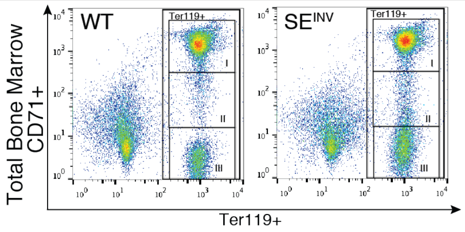

Genotype	n=	Ter119+	I	II	III
WT	5	55.6±6.7	36.6±4	3±1	23.9±4.8
SE^{INV} Hom	5	69.3±4.5	43.6±4.2	7.3±2.8	20.5±2.7

Supplementary Fig. 2

Supplementary Fig. 2 SE^{INV} allele integrity confirmed by optical mapping and homozygous mice are viable with a phenotype reminiscent of stress erythropoiesis. **a** Optical Genome Mapping (OGM) using Bionano shown within ~800Kb window (mm10 chr11:32,880,000-32,600,00) detects the inversion as a structural variant (SV track, marked in a pink box) and otherwise intact genomic region. No other genetic changes were picked up genome-wide. Genes are shown at the top of the tracks as coloured boxes with annotated genes within the α-globin locus. Ref-chr11 is the wildtype reference sequence provided by the Bionano Saphyr software. Allele 1 and allele 2 indicate both alleles in WT and SE^{INV} samples. DNA extracted from wildtype and homozygous SE^{INV} mouse ESCs (clone A4.2. This clone was derived from the heterozygous mESC clone injected in blastocysts to produce the mouse SE^{INV} model). The SE^{INV} homozygous A4.2 clone was also the base clone from which the other SE^{INV} -based genetic models derived from. **b** The observed number of mice resulting from heterozygote (HetxHet) and heterozygote-homozygote (HetxHom) crosses fulfil the expected mendelian ratio (25:50:25). Live homozygote mice confirm no embryonic lethality in SE^{INV} phenotype. **c** A gradual expansion of spleen (splenomegaly) is observed from WT to homozygous SE^{INV} mice. **d** Immunophenotyping using red cell specific surface markers (Ter119 and CD71) show an expansion of the early erythroid compartments (I and II) in SE^{INV}-derived bone marrow from adult mice that have not been exposed to any chemical treatment. There is no perturbation in the mature red cell compartment (III) in SE^{INV}-derived bone marrow compared to that derived from WT.

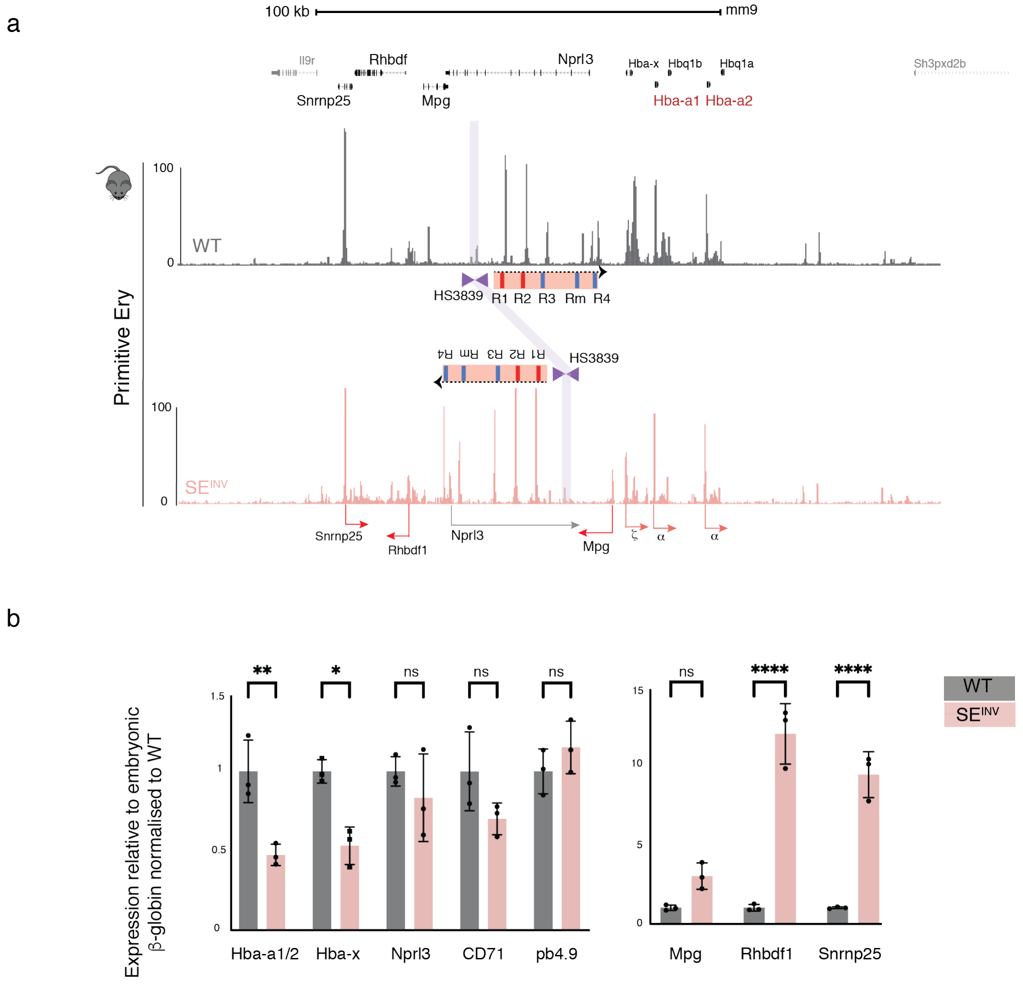

Supplementary Fig. 3

Supplementary Fig. 3 Gene Expression is perturbed at the α-globin locus in the SE^{INV} E10.5 primitive erythroid cells. a ATAC-seq tracks show chromatin accessibility profiles in primary primitive erythroid cells derived from WT (grey) and SE^{INV} (pink) E10.5 embryos. Note the differences in open chromatin profiles corresponding to the genes in the locus. **b** Expression analysis by Real-time qPCR assessing level of mRNA expression for genes of interest relative to the embryonic β-like globin gene (*Hbb-h1*) and WT. Three embryos were analysed from each genotype, biological replicates n=3. The error bars indicate the standard deviation (SD) and black dots represent individual data points. Statistical analysis was performed using two-way ANOVA and Tukey post-hoc test: ****p < 0.0001, **p < 0.004, *p < 0.01, ns: non-significant. Source data is available in the Source Data file.

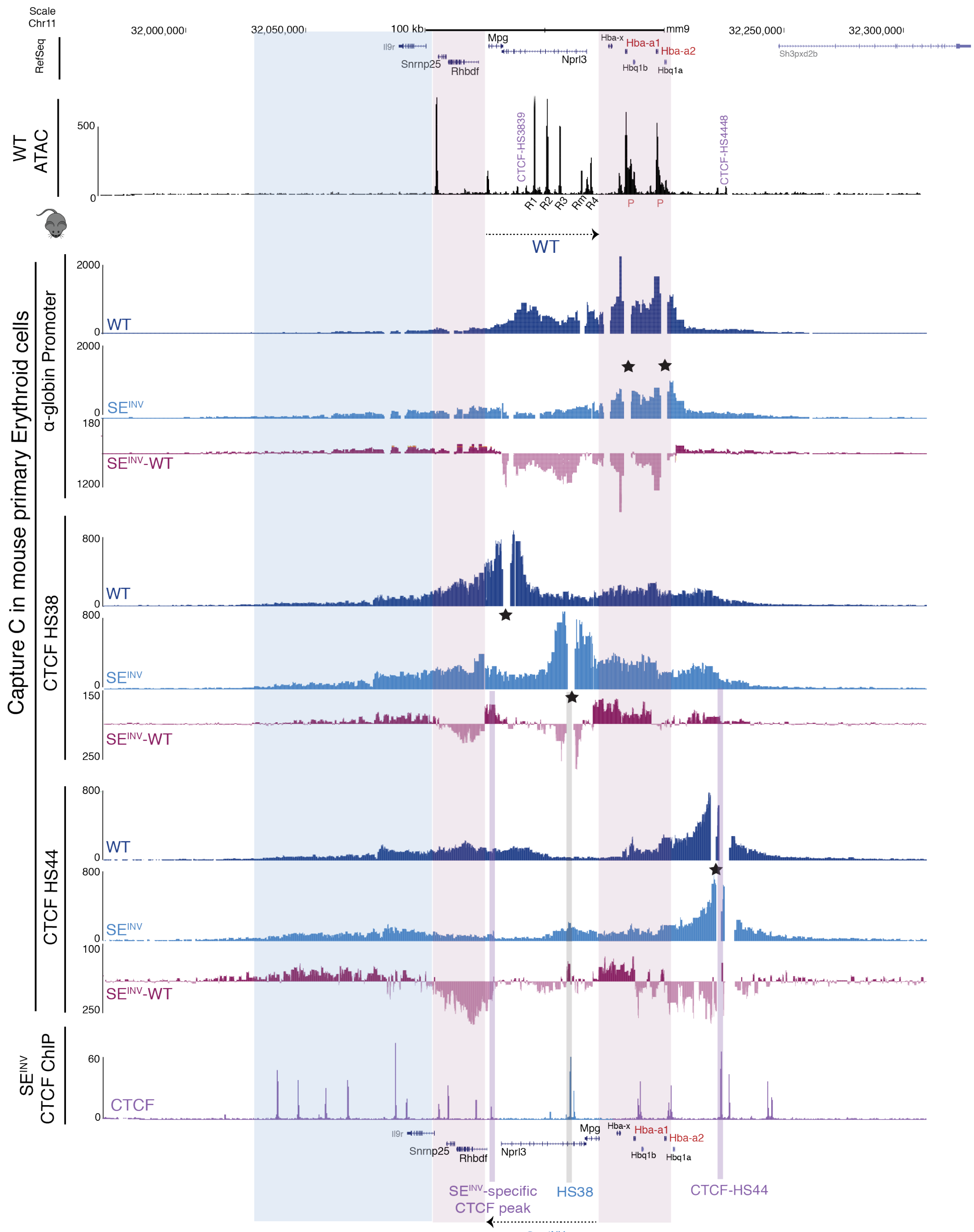

Supplementary Fig. 4

Supplementary Fig. 4 Comparison of NG-Capture C profiles captured from promoters and CTCF sites shows changes in interactions between WT and SE^{INV} primary definitive erythroid cells across the α-globin locus. At the top, scale and RefSeq gene annotation and the bottom the same gene annotation but in the inverted configuration. All the regulatory elements (promoters P, enhancers R1-R4, and CTCF sites) are marked. Top and bottom tracks show normalised (reads per kilobase per million mapped reads, RPKM) and averaged read densities from 3 independent experiments of WT ATAC-seq and SE^{INV} CTCF ChIP-seq, highlighting open chromatin and CTCF occupancy in primary definitive erythroid cells derived from WT and SE^{INV} mice respectively. The NG Capture-C interaction profiles in WT (navy blue) and SE^{INV} (light blue) show means of interacting fragment counts (n=3 independent biological replicates) using a 6 kb window. Additional track shows subtraction (SE^{INV}-WT) per *Dpn*II fragment of significantly interacting fragments using DESeq2 (p.adj<0.05) with light pink for reduced interactions and dark pink for increased interactions in SE^{INV} erythroid cells. The dashed black arrows indicate the direction of the SE in both WT and SE^{INV} models. The stars mark the viewpoints used in the NG Capture-C experiment: the α-globin promoters, 5' CTCF HS38 site highlighted in a shaded grey bar in its new position in SE^{INV}, and 3' CTCF HS44 site and the SE^{INV}-specific CTCF peak highlighted in shaded purple bars. The shaded pink and blue boxes indicate regions of changed interaction profiles observed from various viewpoints in the SE^{INV} model compared to WT.

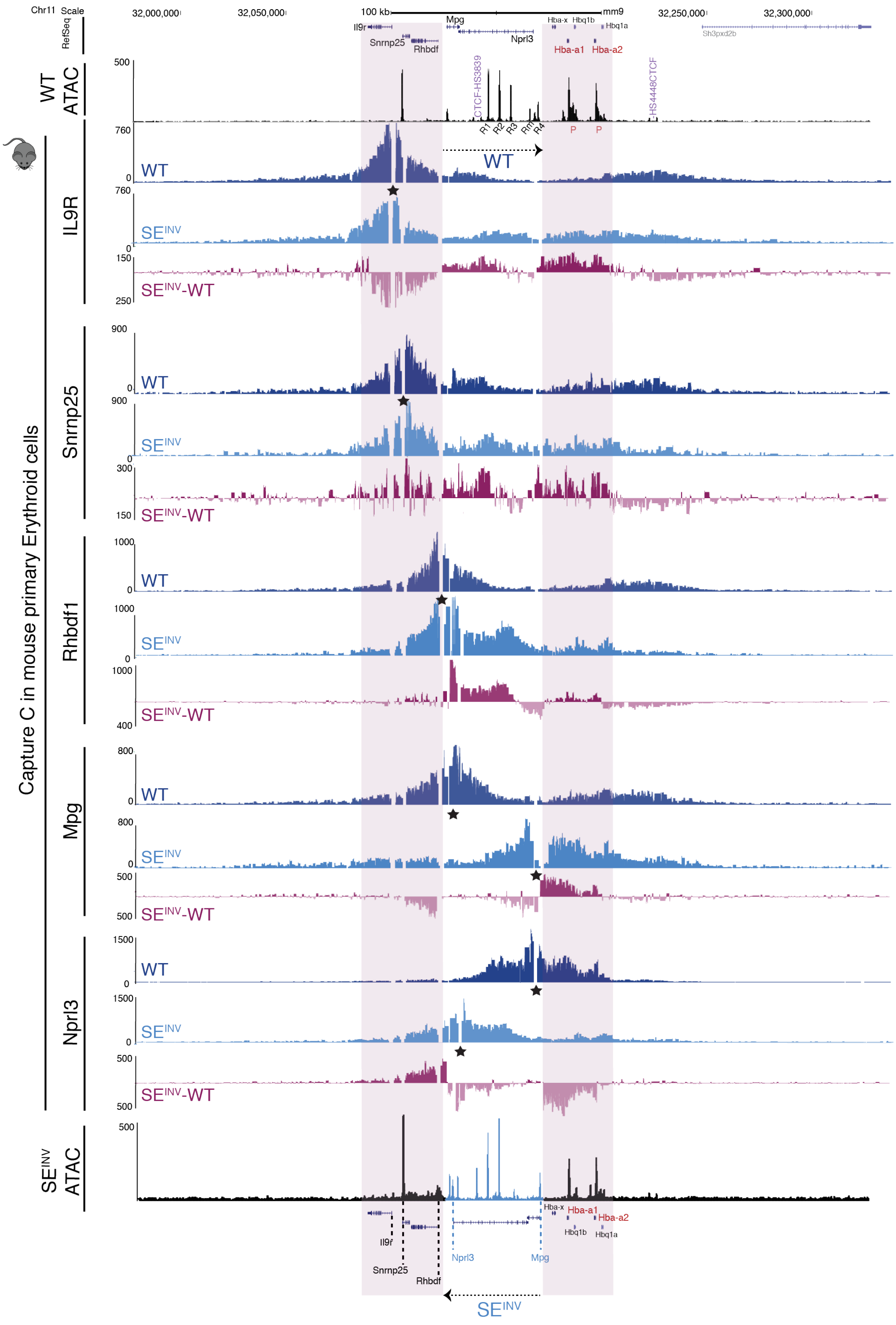

Supplementary Fig. 5

Supplementary Fig. 5 NG-Capture C profiles show redirected interactions from all the promoters present across the α-globin locus when comparing WT and SE^{INV} primary definitive erythroid cells. Same as in Supplementary Fig. 4, except that the bottom track is of an ATAC-seq for the SE^{INV} model and the NG Capture-C viewpoints, as indicated by the stars above the corresponding peaks highlight the promoters of the genes *IL9R, Snrnp25, Rhbdf1, Mpg, Nprl3*, from top to bottom. The shaded pink boxes highlight the regions flanking the inversion specifically the α-globin and the *Rhbdf1* and *Snrnp25* genes. Note the gain and loss of interactions across the α-globin locus.

a

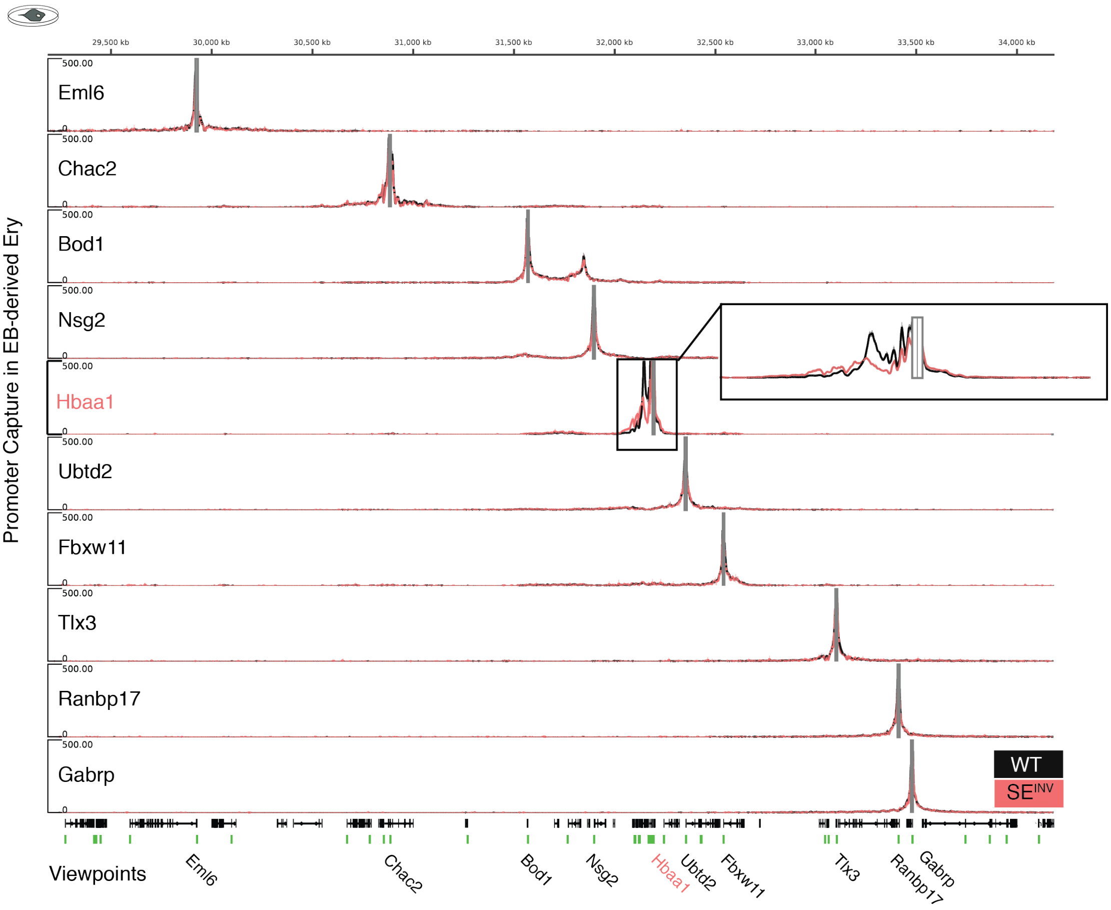

b

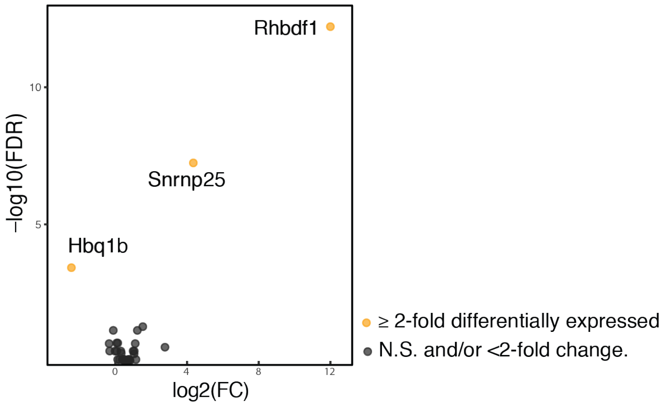

Supplementary Fig. 6

Supplementary Fig. 6 NG-Capture C profiles show undisturbed interactions from all the promoters captured within 5.6Mb flanking the α-globin locus when comparing WT and SE^{INV} primary erythroid cells. a Windowed mean 3C interactions over 4.5 Mb (mm9 chr11:29500000-34000000) for 10 representative promoter NG capture-C viewpoints as indicated, captured with oligonucleotides representing a pool of 42 viewpoints for all the promoters spanning 5.6Mb flanking the α-globin locus. Domain interactions are unaffected in SE^{INV} compared to WT as shown for 9 representative promoter viewpoints except at the *Hba-a2* viewpoint as shown more clearly in the inset panel. Other affected promoter-interactions within the α-globin sub-TAD are shown in Figure S5. Solid lines show means (n=3 independent 3C libraries for each of WT and SE^{INV} primary erythroid cells derived from spleen of adult mice) with one standard deviation (shading) using a 6 kb window. **b** Differential expression between WT and SE^{INV} primary erythroid cells within the 5.6Mb region flanking the α-globin locus; orange dots represent genes with more than 2-fold expression change. The only genes affected within the 5.6Mb are the ones contained in the α-globin sub-TAD (Snrnp25, Rhbdf1 and the pseudo alpha Hbq1). Note that the main α-globin genes (Hba-a1, Hba-a2) are not detected because they were depleted from the RNA-seq material along with ribosmomal genes. PolyA+ RNA-seq data is extracted from the main data set presented in Figure 4D.

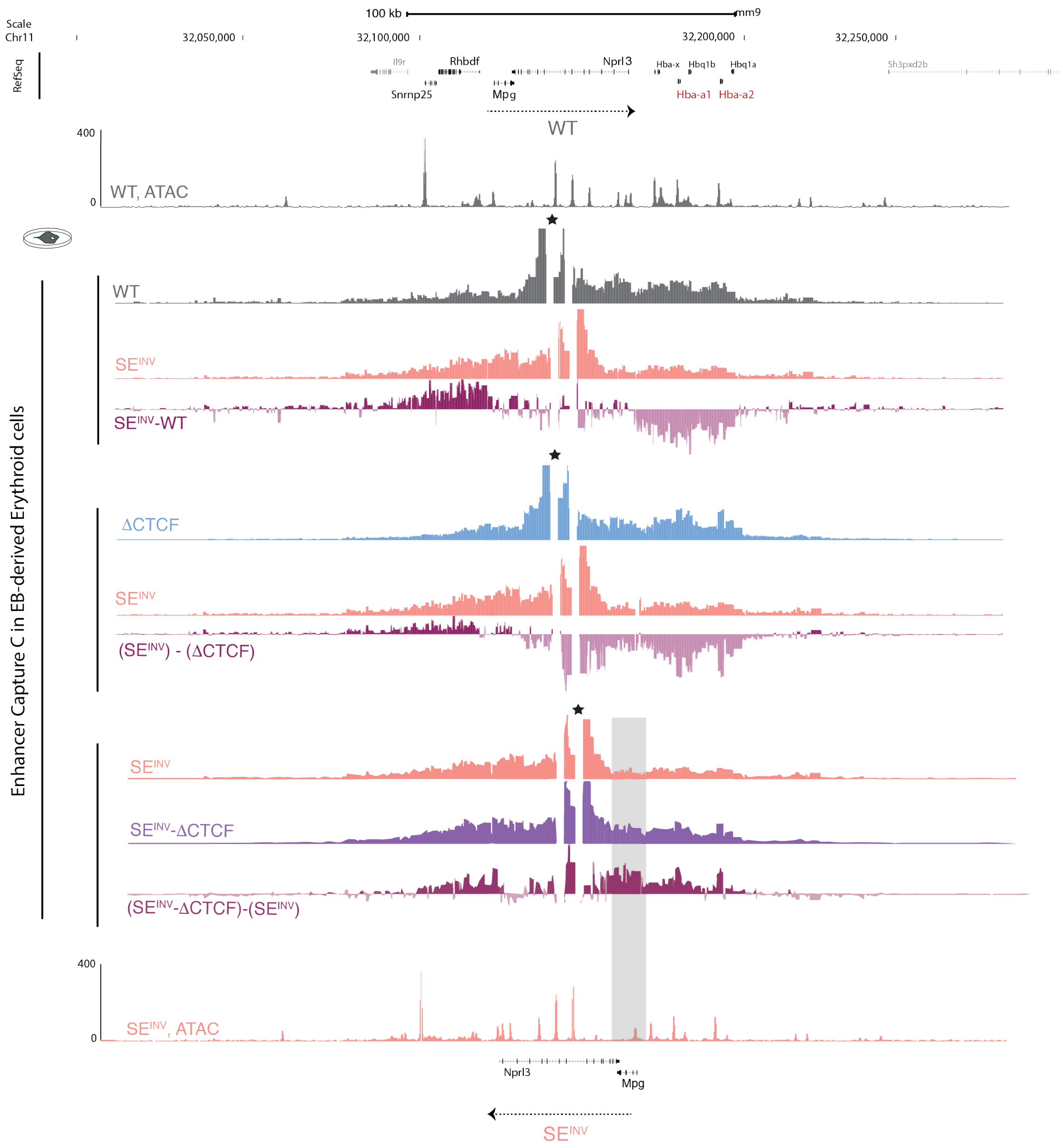

Supplementary Fig. 7

Supplementary Fig. 7 Deletion of the 5' α-globin boundary element (HS3839 CTCF) in SE^{INV} mESC model does not rescue the SE^{INV} phenotype in *in vitro* derived erythroid cells. At the top, scale and RefSeq gene annotation. Normalised (reads per kilobase per million mapped reads, RPKM) and averaged read-densities from 3 independent experiments of ATAC-seq show open chromatin in EB-derived erythroid cells differentiated from WT and SE^{INV} mESCs. NG Capture-C interaction profiles in WT (grey), SE^{INV} (orange), ΔCTCF (light blue) and SE^{INV}-ΔCTCF (purple) show means (n=3 independent biological replicates) of interacting fragment count using a 6 kb window. Additional track shows subtractions (SE^{INV} -WT, (SE^{INV}) - (ΔCTCF), (SE^{INV}-ΔCTCF) - (SE^{INV})) per *Dpn*II fragment of significantly interacting fragments using DESeq2 (p.adj<0.05) with light pink for reduced interactions and dark pink for increased interactions across the models analysed. The grey shaded area, encompassing the *Mpg* gene located between the SE and the α-globin genes in the SE^{INV} models, indicates the increased interactions at the newly positioned *Mpg* in the SE^{INV}-ΔCTCF. The dashed black arrows indicate the direction of the SE in both WT (top) and SE^{INV} (bottom) models. The stars mark the viewpoint (R1 enhancer) used in the NG Capture-C experiment.

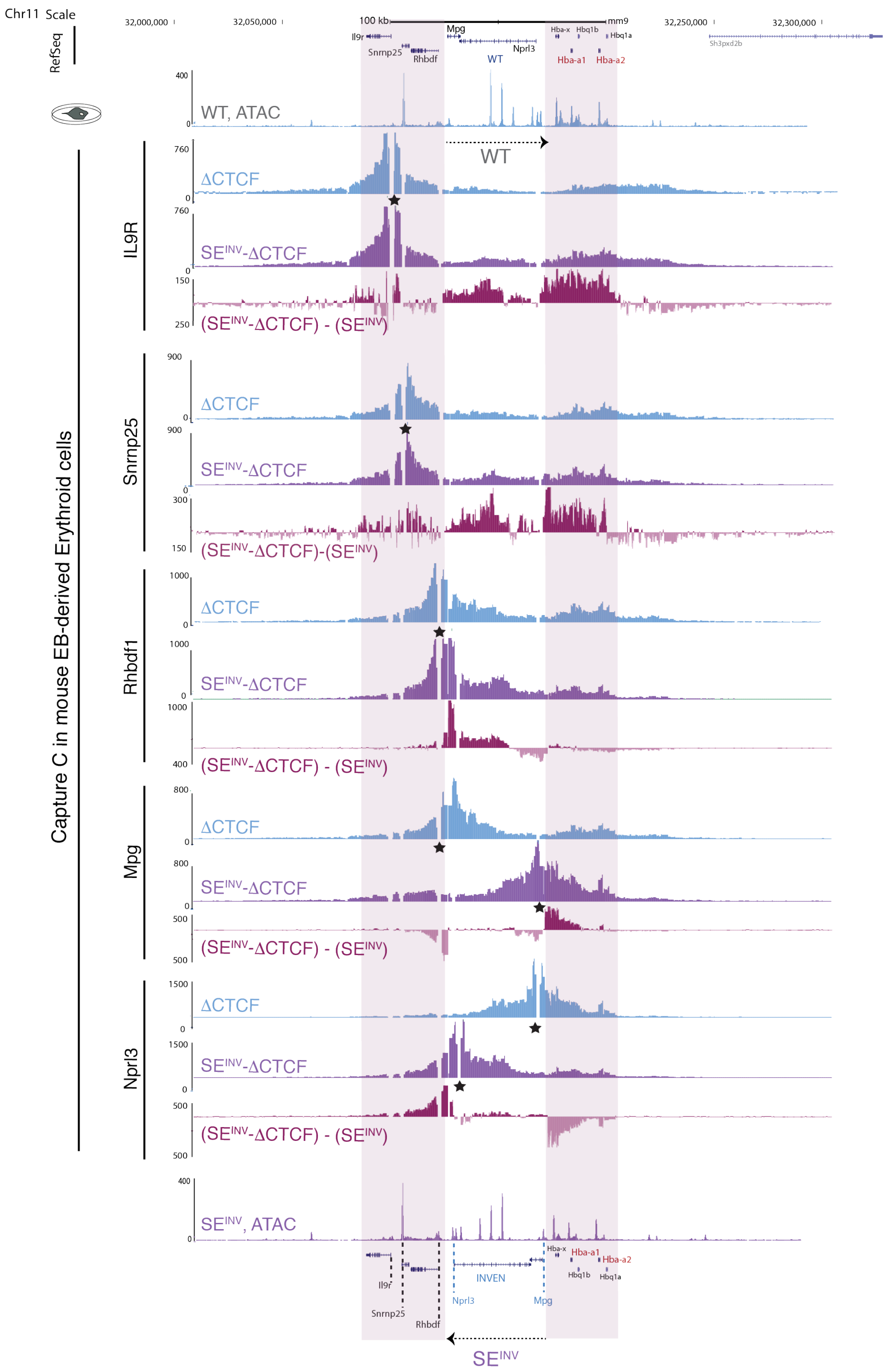

Supplementary Fig. 8

Supplementary Fig. 8 NG-Capture C profiles captured from all the promoters present within the α-globin sub-TAD show changes in interactions when comparing α-globin 5' boundary (HS3839) knock out (ΔCTCF) and HS3839 knock out in the SE inversion model (SE^{INV}-ΔCTCF) in EB-derived erythroid cells. Same as in Supplementary Fig. 5 except; the data is from ΔCTCF and SE^{INV}-ΔCTCF EB-derived erythroid cells.

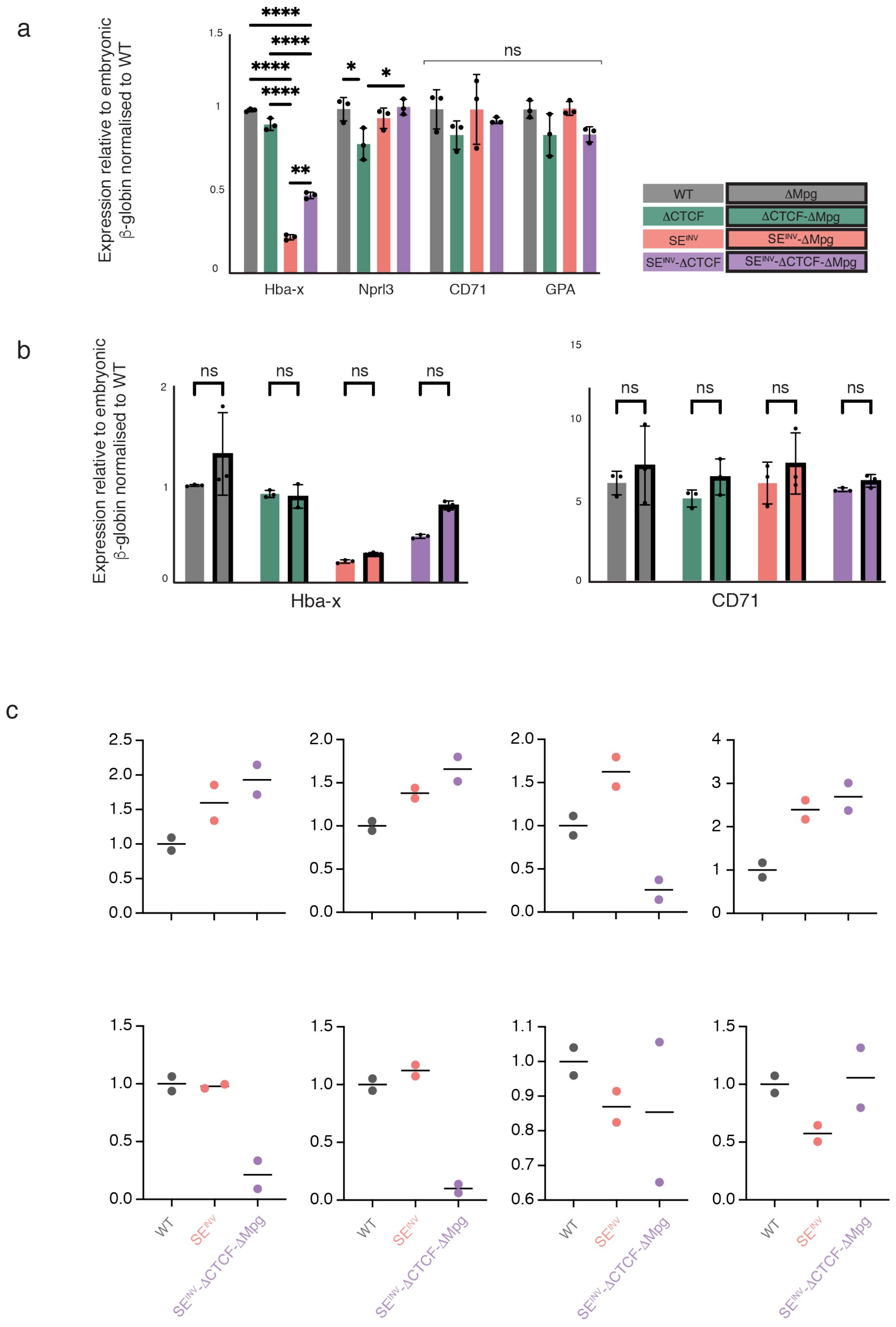

Supplementary Fig. 9

Supplementary Fig. 9 Expression analysis and Rad21 ChIP quantitation at the α-globin locus in erythroid cells derived from WT, SE^{INV}, SE^{INV}-ΔCTCF and SE^{INV}-ΔCTCF-ΔMpg mESC models. **a** Gene expression analysis by real-time qPCR assessing levels of mRNA for controls *Nprl3, CD71, pb4.2*, and the embryonic α-like globin (*Hba-x*) relative to the embryonic β-like globin gene (*Hbb-h1*). Independent erythroid differentiation experiments were analysed for each model, biological replicates n=3. The error bars indicate the standard deviation (SD) and black dots represent individual data points. Statistical analysis was performed using one-way ANOVA and Tukey post-hoc test: ****p < 0.0001, **p < 0.004, *p < 0.04, ns: non-significant. **b** Same as above for the embryonic α-globin (*Hba-x*) and red cell marker (CD71) relative to the embryonic β-like globin gene (*Hbb-h1*). Statistical analysis was performed using one-way ANOVA and Tukey post-hoc test, ns: non-significant. **c** Rad21 ChIP-seq read counts from WT, SE^{INV}, and SE^{INV}-ΔCTCF-ΔMpg EB-derived erythroid cells. The plots show the mean of two biological replicates (n=2) and the black bar indicates the mean. Data shown for the following regions: *snrnp25* promoter, *Rhbdf1* CTCF site, *Mpg* promoter, *Rhbdf1* promoter, HS38 CTCF site, HS39 CTCF site, R2 enhancer, and *Hba* promoter, normalised to the average number of reads over selected peaks in the β-globin locus. Source data is available in the Source Data file.

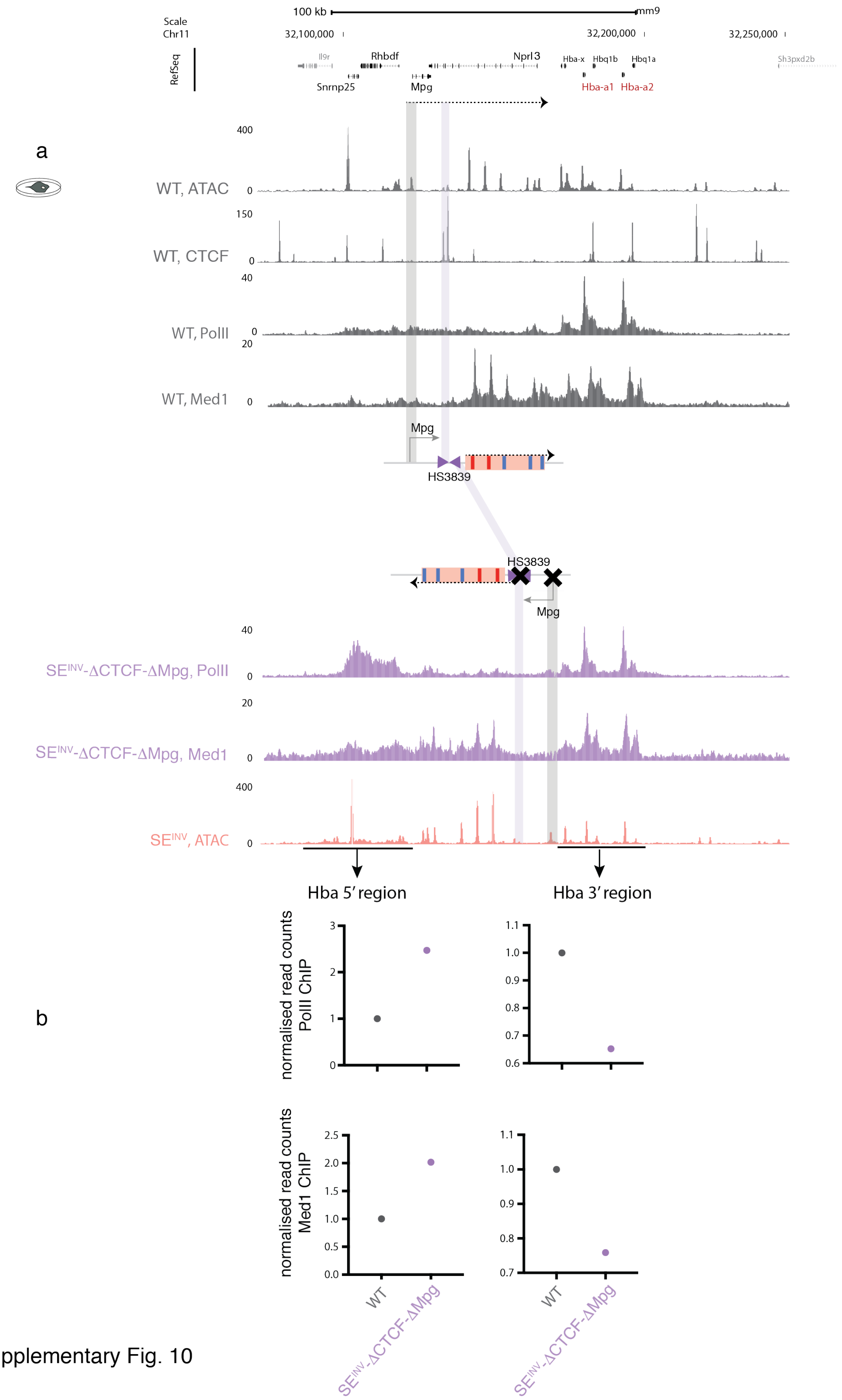

Supplementary Fig. 10

Supplementary Fig. 10 PolII and Med1 ChIP at the α-globin locus in erythroid cells derived from WT and SE^{INV}-ΔCTCF-ΔMpg mESC models show a distribution pattern that mirrors the SE sequence and functional orientation. a Top two tracks: RPKM-normalised ATAC-seq and CTCF ChIP-seq tracks for WT EB-derived erythroid cells. Below, representative RPKM-normalised PolII and Med1 ChIP-seq tracks for WT and SE^{INV}-ΔCTCF-ΔMpg EB-derived erythroid cells, Bottom: ATAC-seq track for SE^{INV} EB-derived erythroid cells for orientation. The schematic represents the WT (top) versus the SE^{INV}-ΔCTCF-ΔMpg (below) configurations for orientation. The grey and purple highlighted areas indicate the *Mpg* gene and HS3839 CTCF sites respectively. **b** PolII and Med1 ChIP read counts in erythroid cells derived from WT and SE^{INV}-ΔCTCF-ΔMpg *in vitro* differentiated mESCs (n=1). Data shown for the regions 5' and 3' of the inverted SE and marked by a black line below the SE^{INV} ATAC-seq track. The plots show the one data point for each sample. Reads are normalised to the average number of reads over a selected region in the β-globin locus.

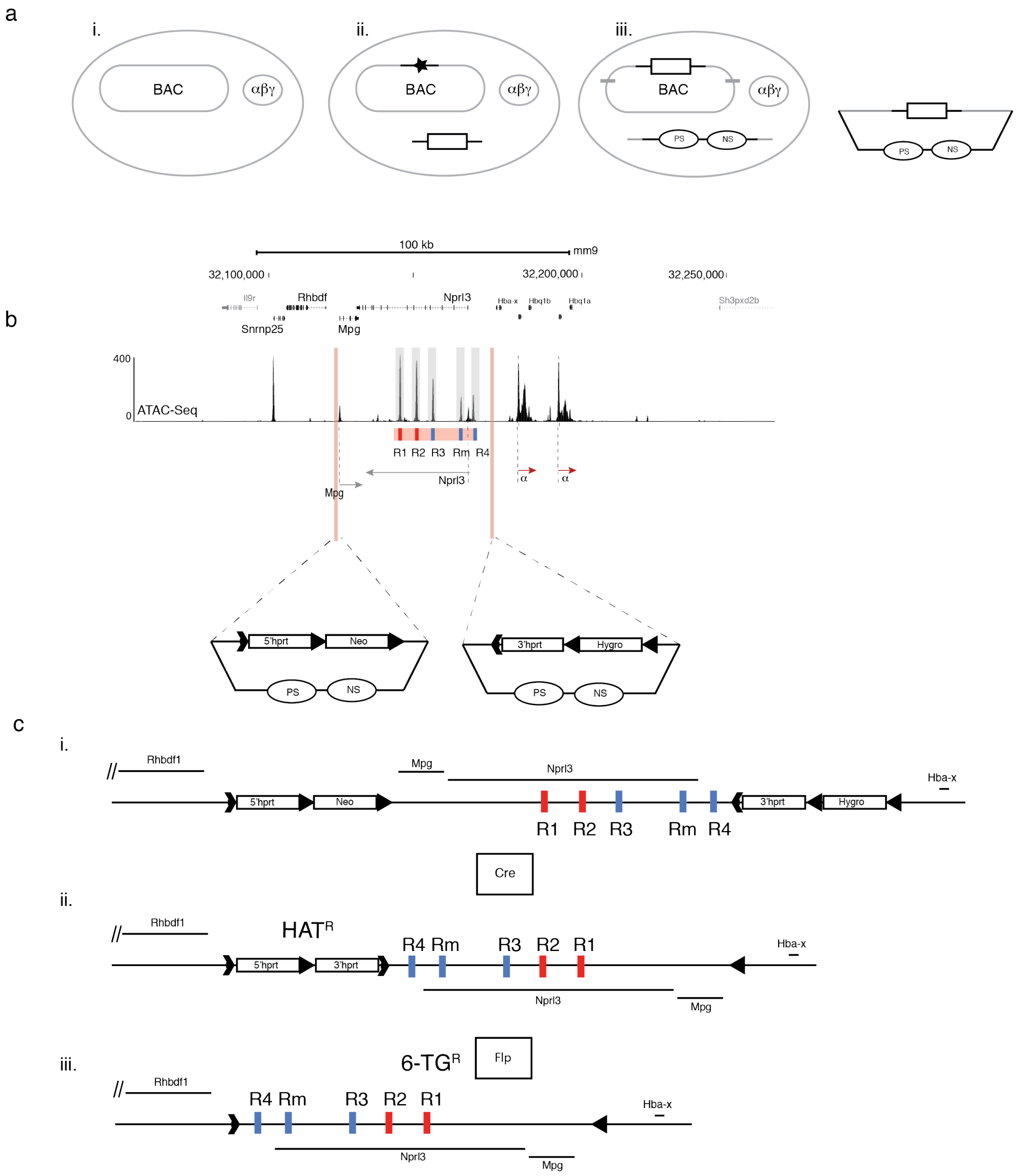

Supplementary Fig. 11

Supplementary Figure 11. Overview of the α-globin super-enhancer inversion genome editing strategy. a Targeting vector construction using the recombineering methodology. i) E.Coli harbouring a BAC encompassing the α-globin locus and derived from 129/Ola mouse strain, including sequences homologous to the sequences to be targeted in E14 mESCs for LoxP insertion (upstream and downstream of Mpg and Nprl3 genes respectively) and phage-derived proteins (αβγ) necessary for target DNA alteration by homologous recombination. ii) Insertion of the LoxP sequences in the designated location in the locus (black star) using a selection cassette flanked by homology sequences to the insertion site (white box with 2 black lines). iii) Targeting vector assembly by retrieving the modified sequence using a minimal vector with positive (PS) and negative selection (NS) markers and homology sequences (grey lines) to the boundary of the homology arms required to target the locus in ES cells. **b** LoxP insertion at two positions in the α-globin locus in mESCs. Schematic representation of the mouse α-globin locus and upstream region (genes and enhancers annotated as in Fig. 1-b). Two targeting vectors harbouring arms of homology (3 to 5 Kb) to sequences flanking the LoxP insertion sites. Selection cassettes flanked by LoxP and Frt sites in opposite directions are used. **c** Cre-mediated inversion of the region encompassing the enhancers. i) Schematic representation of a targeted allele in cis, ii) the inversion of the locus and deletion of positive selection cassettes upon Cre-recombinase expression (selected for by the restoration of the *Hprt* gene expression, HATR), iii) FLP-mediated deletion selected for by resistance to 6-TG upon deletion of the reconstructed *Hprt* mini-gene.

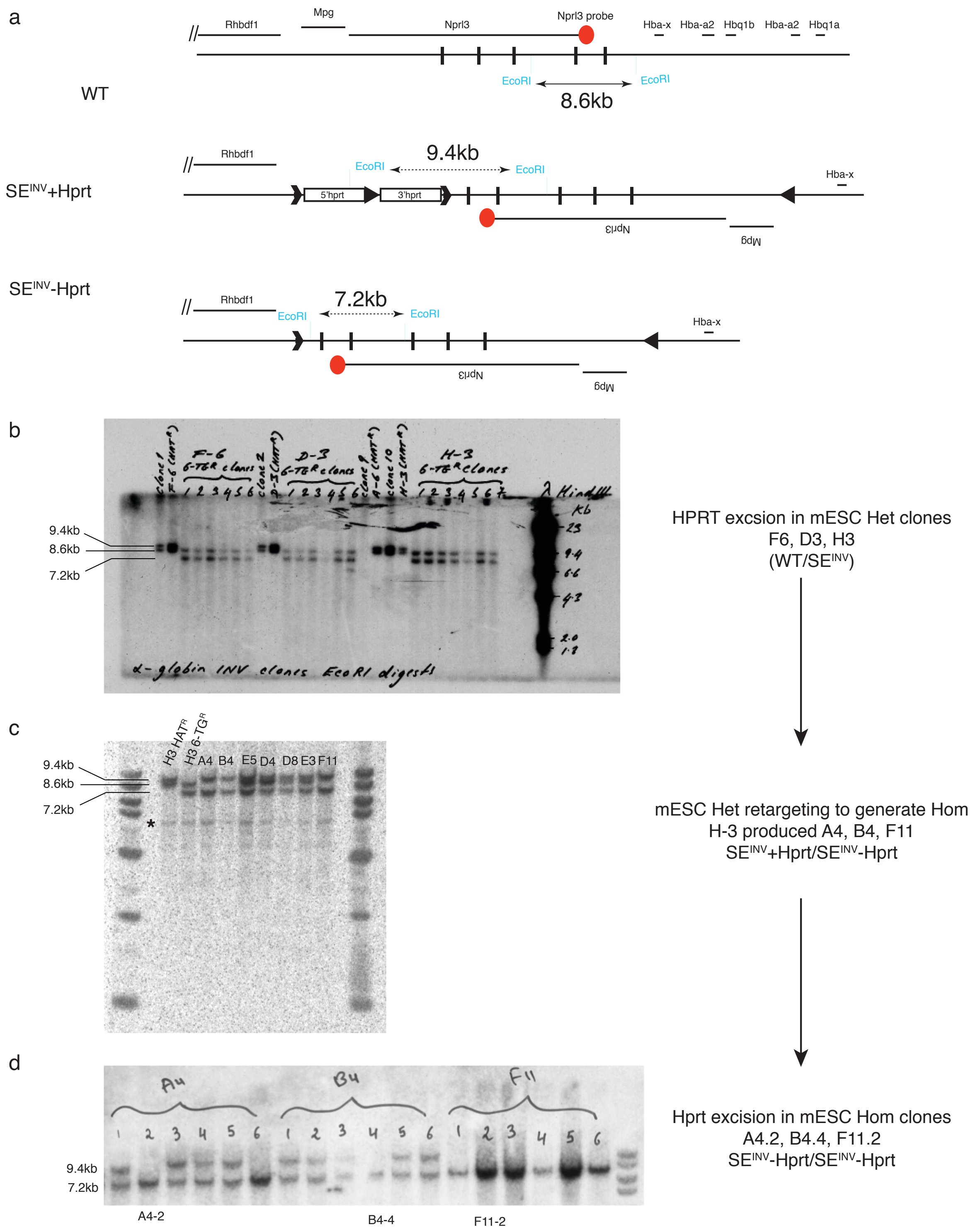

Supplementary Fig. 12

Supplementary Figure 12. Southern blot data showing heterozygous (WT/ SE^{INV}) and homozygous SE^{INV} (SE^{INV} / SE^{INV}) mESCs clones before and after *Hprt* minigene excision. a Schematic representation of the Southern Blot screening strategy for *Hprt* minigene excision upon FLP expression. The horizontal black line indicates the genomic region spanning the α-globin locus with genes annotated on top and marked by smaller horizontal black lines. The red circle indicates the location of the probe designed within the Nprl3 gene. Vertical boxes indicate the 5 enhancer-like elements (R1, R2, R3, Rm, R4 from 3' to 5'). EcoRI restriction enzyme digestion fragment is designated by a double-arrow line (8.6kb fragment in WT, 9.4Kb in SE^{INV} allele with Hprt minigene intact (SE^{INV} +Hprt), 7.2Kb in SE^{INV} allele with *Hprt* minigene excised (SE^{INV} -Hprt)). **b** Southern blot confirming *Hprt* minigene excision in 3 independent mESCs heterozygous for SE^{INV} (WT/ SE^{INV}); F6, D3, H3. Note the change in the size of the bands from 9.4kb (unexcised) to 7.2Kb (excised) in 6 subclones for each of the three clones. Lambda phage DNA digested with HindIII determined the large genomic fragment sizes (ladder to the right of the blot). **c** Southern blot confirming the generation of homozygous mESC clones from a heterozygous WT/ SE^{INV} clone, already *Hprt* excised (H3- 6TG^{R}). The resulting homozygous mESCs (example A4, B4, F11) harbour one SE^{INV} allele with excised *Hprt* and one SE^{INV} allele that still retains the *Hprt* allele (SE^{INV} +Hprt/ SE^{INV} -Hprt) as indicated by the size of the detected bands (9.4Kb and 7.2Kb respectively). **d** Southern blot confirming *Hprt* minigene excision in 3 independent mESCs (A4.2, B4.4, F11.2) homozygous for SE^{INV} (SE^{INV} / SE^{INV}).

Supplementary Tables

Supplementary Table 1. Sequences for Southern Blot probes and Sanger Sequencing primers used for screening the SE^{INV} models

**Southern Blot probes**	
Mpg probe primer pair	F: AGTGGAGAGGCTCAGAAGG
	R: TCTCTGTCTTGGCCTTCC
Nprl3 probe primer pair	F: CATGATTGTTATGTGATGAGC
	R: TTTTATTTCATGGGCTGGAG

Supplementary Table 2. gRNAs used for CRISPR-Cas9 genome editing

**Modification**	**sgRNA Sequence**
ΔR1	GTGACCATAAGTTGATTAG
	GTACATACGCCGGGCGTGG
ΔR2	TACCTCCAAGGTTTTGCTC
	GCCGTGACACTTCATGCTCA
D3839 (double nickase)	GGCCACTGGGGGCGCCATTC
	CGCCATTAAAAGGTCCTGCT
	TTGAGCAAACTGAGGTCCTG
	AGGCCTCTGCTACCCTCTGG
MpgKO	GGATGGCAATCGGGCCATT
	GGCCTGTACTTGTGAAGTCGA

Supplementary Table 3. Primer pairs used for PCR screening of mESC targeted clones

ΔR1 screening	Over deletion (short amplicon)
	F: TGTGTGTACAGTTTCCAGGAGGG
	R: CTTCCCTTAGGAAGGGATCTCCC

	Over deletion (long amplicon)
	F: TTTCGGGAGCTGGGAGTAGA
	R: TTGAACCGTTAAACGCAGCAG
	R1 internal
	F: GGGCACAGCAAAAGAGGAAA
	R: AATGGTCCTTGTCCTCCCAG
R2^{INV} screening	Over inversion breakpoint
	F: AGGACTTCCTCTCCAGTACCC
	R: CCCCCAAGTATTACTCAGCAG
	F: CAGATTACCACTAGGCTCCTG
	R: CTGAGGATGACCTAGAATTCC
	R2 internal
	F: CTCCTGGCCCATGTCTCTC
	R: TAACAGGCACAGGGTCACTT
ΔCTCF screening HS38 ScaI integration (short)	F: GGCGTTCAGGGACAGTGAGCACAT
	R: AAGTGTGTGTGGCAGCCCTTTGGG
ΔCTCF screening HS39.5 ScaI integration (short)	F: ACAGCAACCATCTGGGTGAG
	R: TGCTGGTGTCTGTGGACAAG
ΔCTCF screening HS38 ScaI integration (long)	R: GGGAAGCACAATGAGCTCCT
	R: AGCATGTGGGAAGTGAGAGC
ΔCTCF screening HS39.5 ScaI integration (long)	R: ACCAGCCTCTAATTGCCACC
	R: TGCAGGGTCAGGTAGGATGA
ΔMpg screening for ScaI integration	F:CACCATTCAGAGATAAGGTTGGG
	R: AGCAAGGAAATCTGGCCACATCC

Supplementary Table 4. Flow cytometry and magnetic column purification antibodies

Protein target Antibody, source	Source	Working concentration
FITC Rat Anti-Mouse CD71	eBioscience 11-0711-85	2.5 μg/ml
PE Rat Anti-Mouse Ter119	BD Pharmingen 553673	2 μg/ml
Hoechst	Invitrogen H3569	1 μg/ml

Supplementary Table 5. Antibodies used for ChIP-seq

Protein target - Antibody	Source	Working concentration
H3K27ac	Abcam ab4729	0.5 µg/ml
H3K4me1	Abcam ab8895	0.3 µg/ml
H3K4me3	Abcam 8580	2.5 µg/ml
H3K27me3	Cell Signaling 9733	2 µg/ml
CTCF	Merck Millipore 07-729	5 µg/ml
Rad21	Abcam ab154769	5 µg/ml
Anti-Med1	Bethyl A300-793A (Lot 11)	5 ug
Anti-Rpd1-NTD	Cell signalling 14958 (D8L4Y)	5 ug / ~250,000 cells

Supplementary Table 6. Primer pairs used for expression analysis by RT-PCR

**Gene**	**Primer**	**Sequence**
Hba-a1/2	Forward	CTGGGGAAGACAAAAGCAAC
	Reverse	GCCGTGGCTTACATCAAAGT
Hba-x	Forward	ATGCGGTTAAGAGCATCGAC
	Reverse	GGGACAGGAGCTTGAAGTTG
Hbb-b1/2	Forward	ACGATCATATTGCCCAGGAG
	Reverse	ATGGCCTGAATCACTTGGAC
Hbb-bh1	Forward	TGGACAACCTCAAGGAGACC
	Reverse	ACCTCTGGGGTGAATTCCTT
Hbb-y	Forward	TGGCCTGTGGAGTAAGGTCAA
	Reverse	GAAGCAGAGGACAAGTTCCCA
Rn18s	Forward	GTAACCCGTTGAACCCCATT
	Reverse	CCATCCAATCGGTAGTAGCG
pb4.2	Forward	GCTCCAACCCACACATTTCT
	Reverse	GCATCTCTTTCCTCCACTGC
CD71	Forward	TCCGCTCGTGGAGACTACTT
	Reverse	ACATAGGGCGACAGGAAGTG

Nprl3	Forward	ATGTTCGCCAGTGTTGTTGA
	Reverse	GCTCTTCAGGTACCCCTTCC
Mpg	Forward	CTTCTCCAGCCCAGAGGAC
	Reverse	ATGCCTCAGTCTCCACAATG
Rhbdf1	Forward	CGGCCACTTGGTGATATCTT
	Reverse	ATCCTAGTGCCCCAGACCTT
Snrnp21	Forward	GAGGTAATGCCTGTGGTCGT
	Reverse	GGTCAGATGGTATGTCCGCC
