# Supplementary material for: The α-globin super-enhancer acts in an orientation-dependent manner
Source: Nat Commun. 2025 Jan 25;16:1033. doi: 10.1038/s41467-025-56380-1 (PMC11762767; doi:10.1038/s41467-025-56380-1)
Supplement: Supplementary file 3 — Description of Additional Supplementary Files [file 41467_2025_56380_MOESM3_ESM.pdf]

Description of Additional Supplementary Files

File Name: Supplementary Data 1
Description: A PDF file containing Southern Blot and PCR strategy and data for sequential targeting and screening of mESCs to generate the alpha-globin SE inversion.
