# Supplementary material for: The α-globin super-enhancer acts in an orientation-dependent manner
Source: Nat Commun. 2025 Jan 25;16:1033. doi: 10.1038/s41467-025-56380-1 (PMC11762767; doi:10.1038/s41467-025-56380-1)
Supplement: Supplementary file 4 — Supplementary Data 1 [file 41467_2025_56380_MOESM4_ESM.pdf]

Targeted Clones Screening Strategy

3’ side of inversion

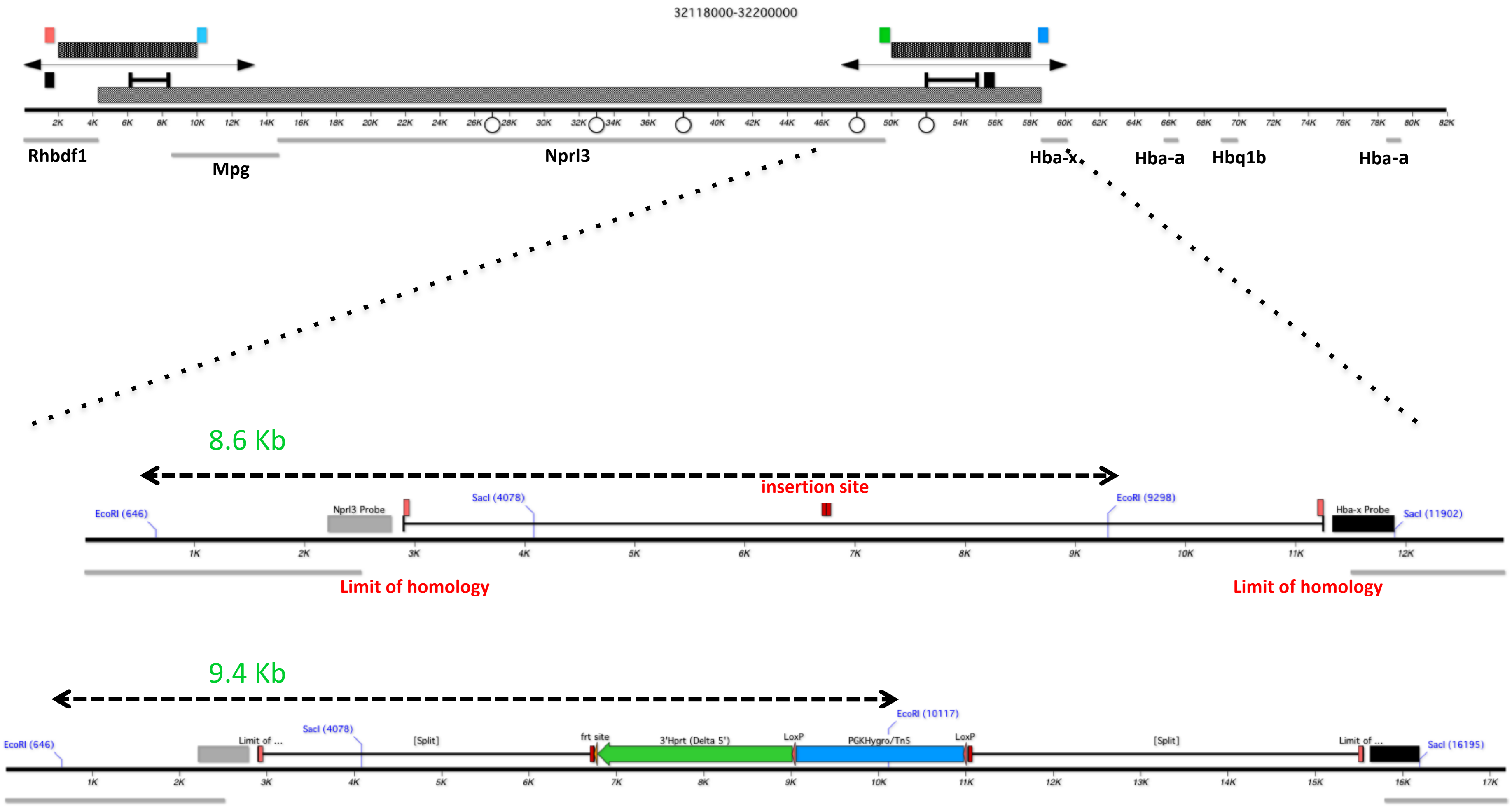

85% clone DNA recovery (153/180)
39% positive (60/153)

Expected band size: Targeted 9.4 Kb,
WT 8.6 Kb

Plate 1

3084
35%+/

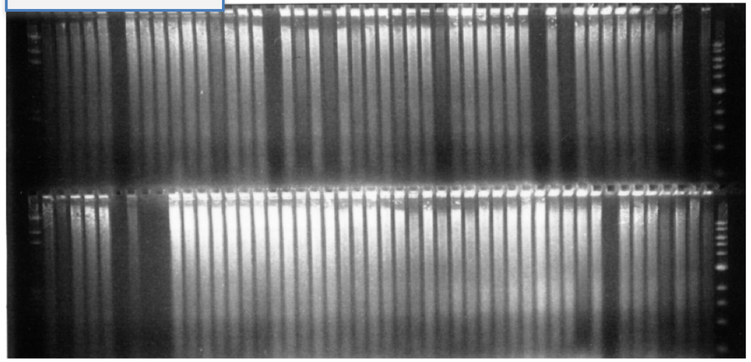

Plate 2

30+/69
43%

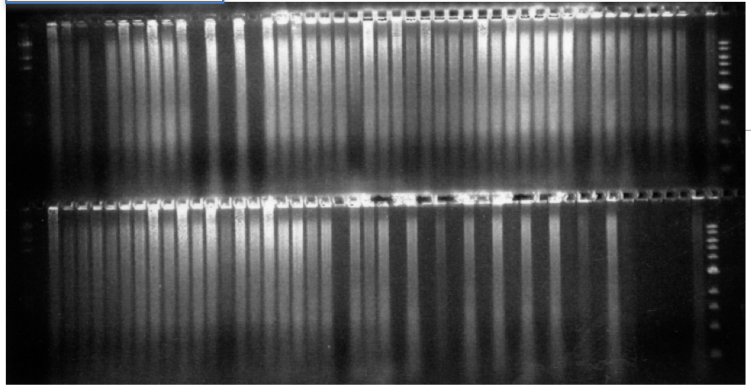

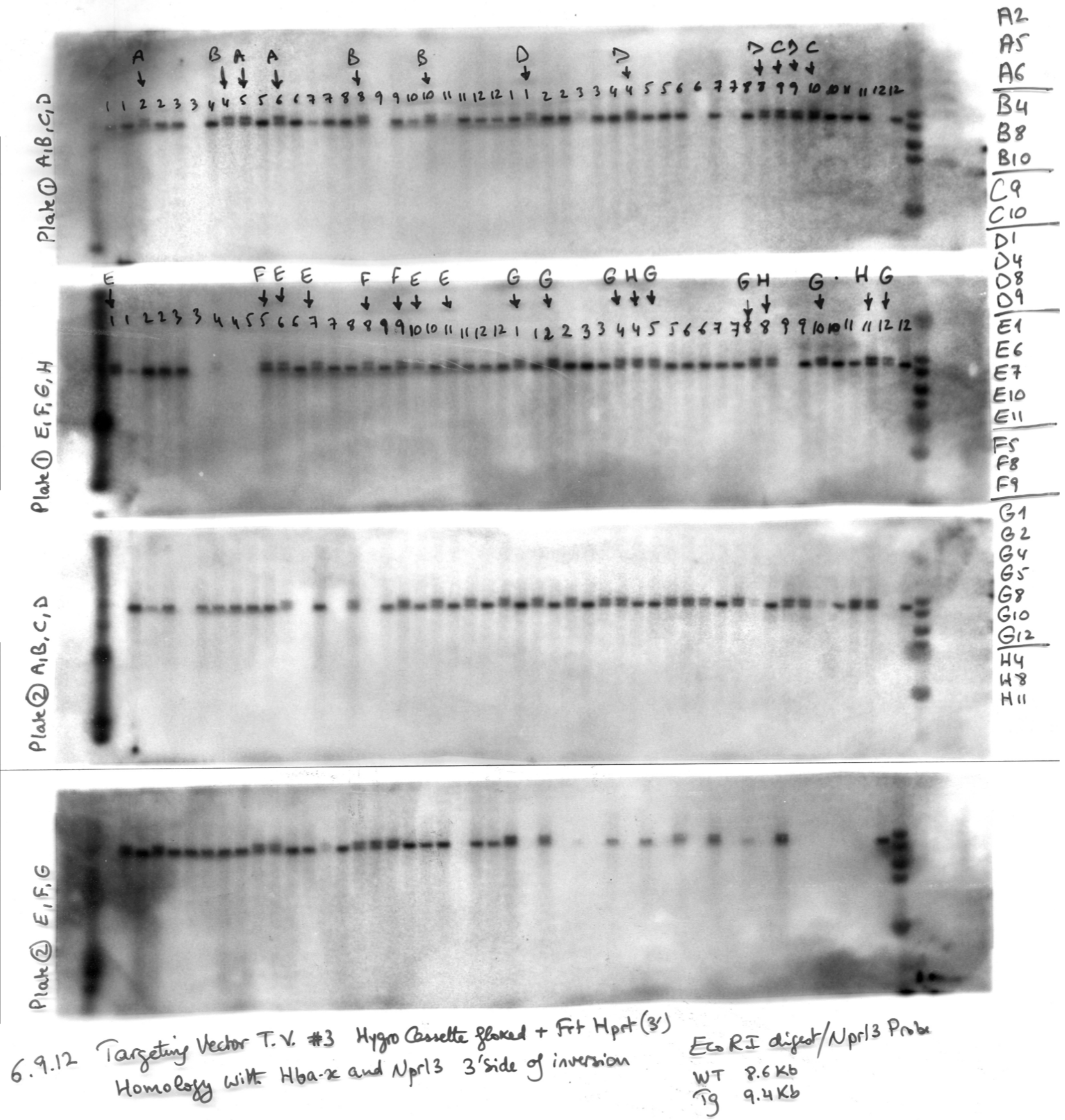

General overview of the targeting protocol

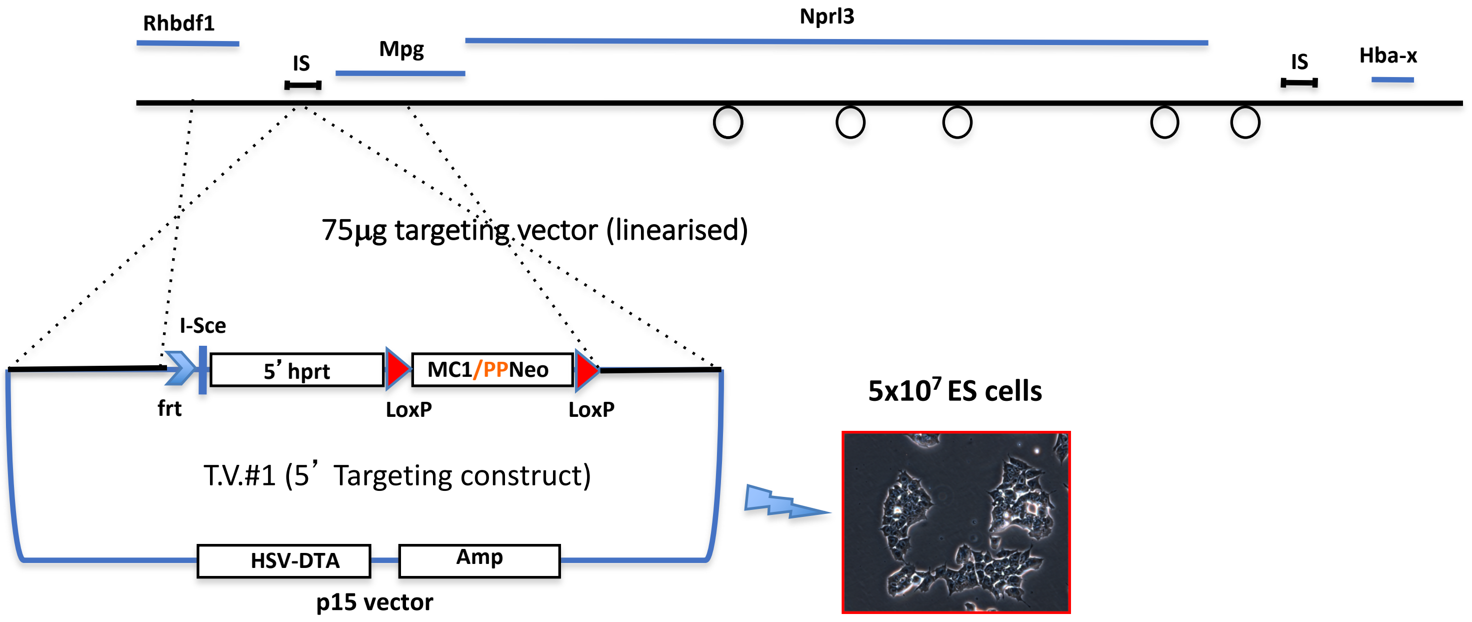

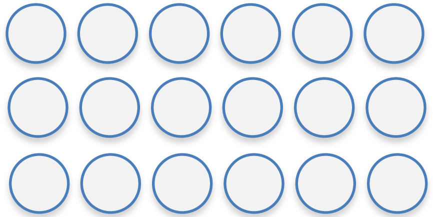

Neomycin added 2 days later

100's of colonies grew!

8 plates picked, 768 clones. 348 clones screened by Southern Blots

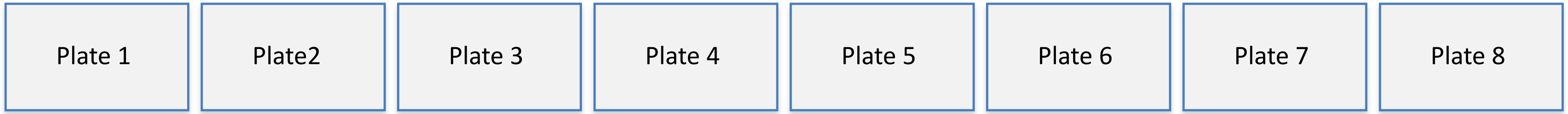

Screening Strategy

5' side of inversion

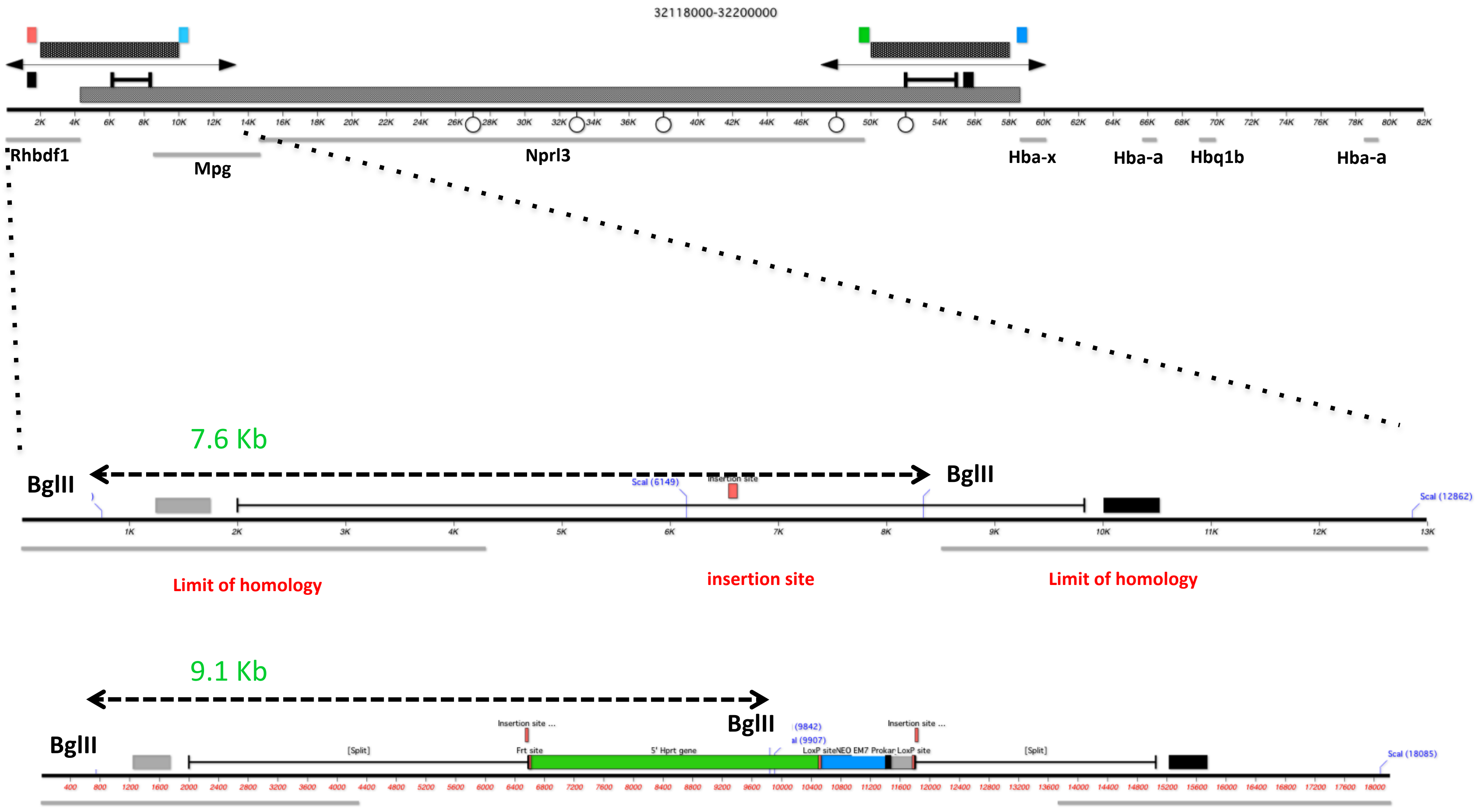

67% clone DNA recovery (259/384)
53% positive (138/259)

Targeted 9.1 Kb
WT 7.6 Kb

Plate 5

38+/57
66% +

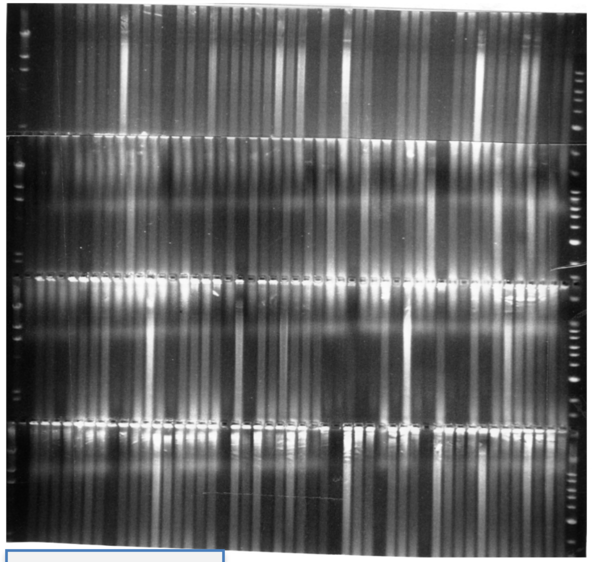

Plate 6

37+/61
60% +

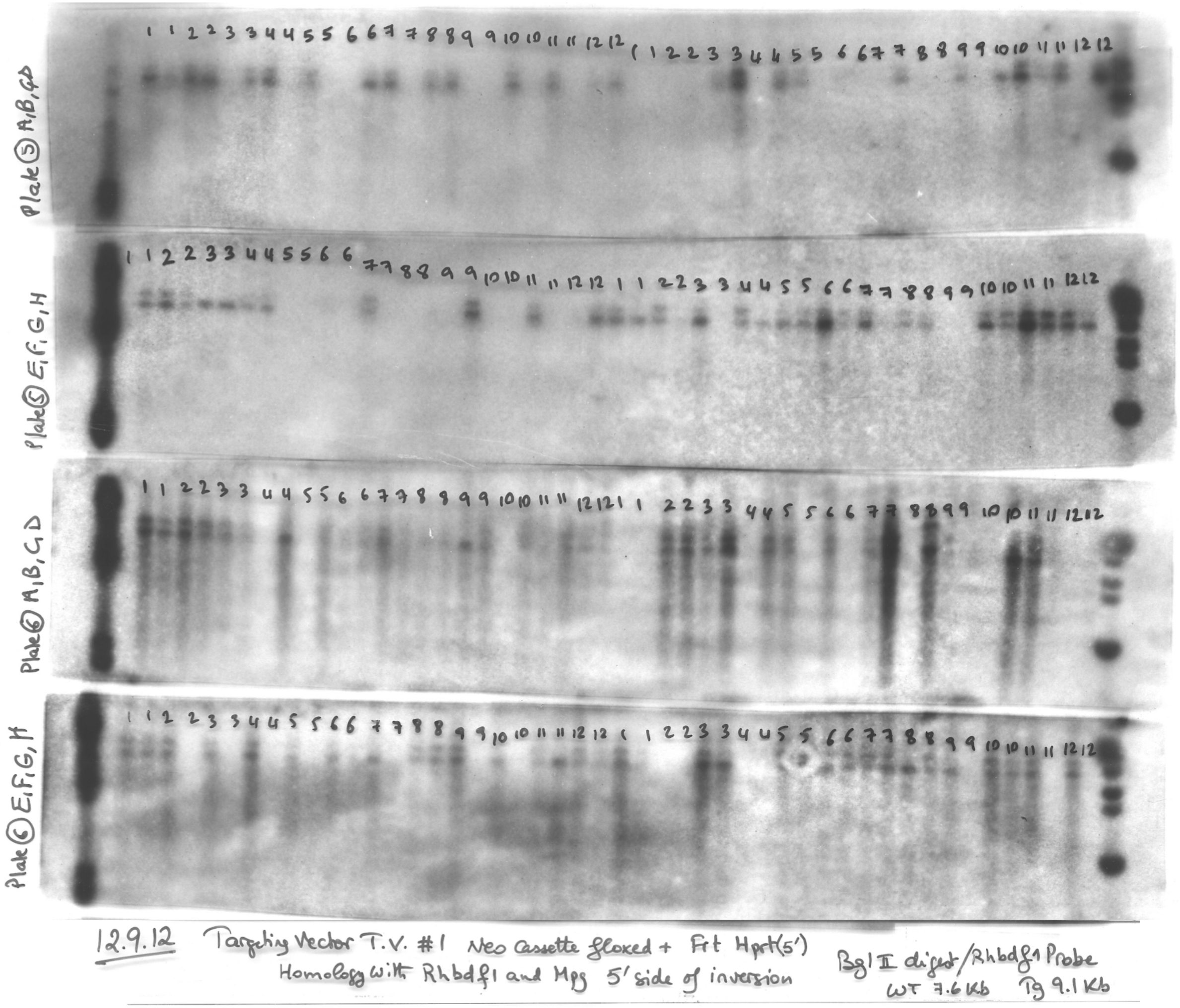

Targeted 9.1 Kb
WT 7.6 Kb

Plate 7

37+/72
51% +

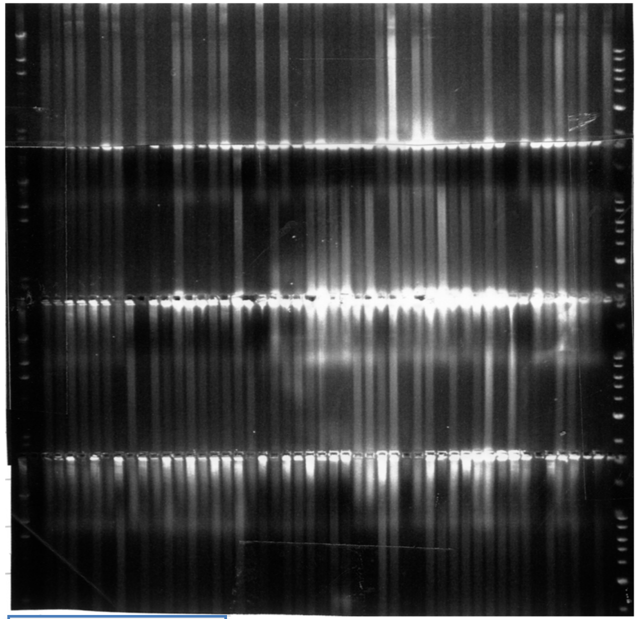

Plate 8

26+/69
37% +

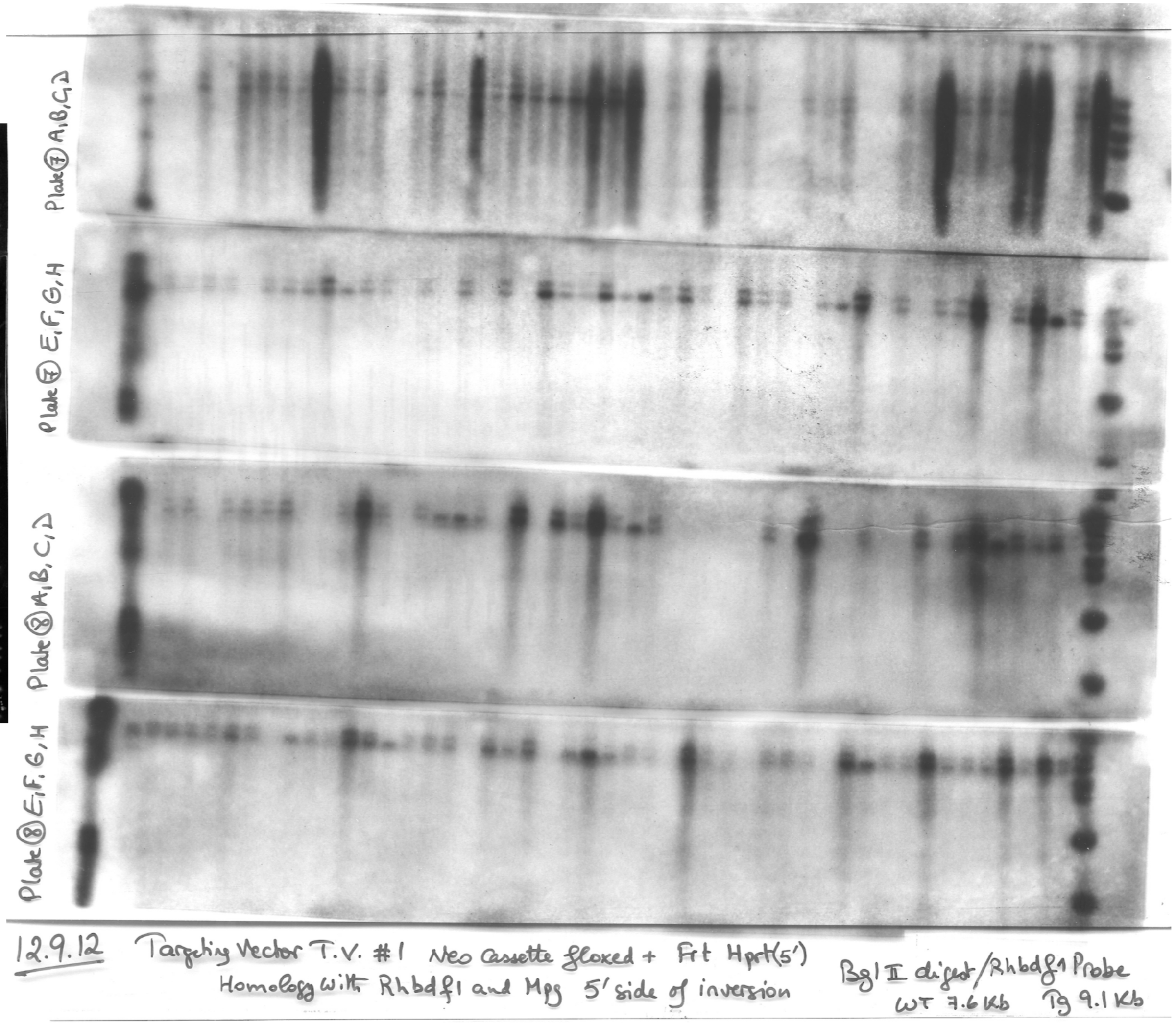

Double targeting in Cis

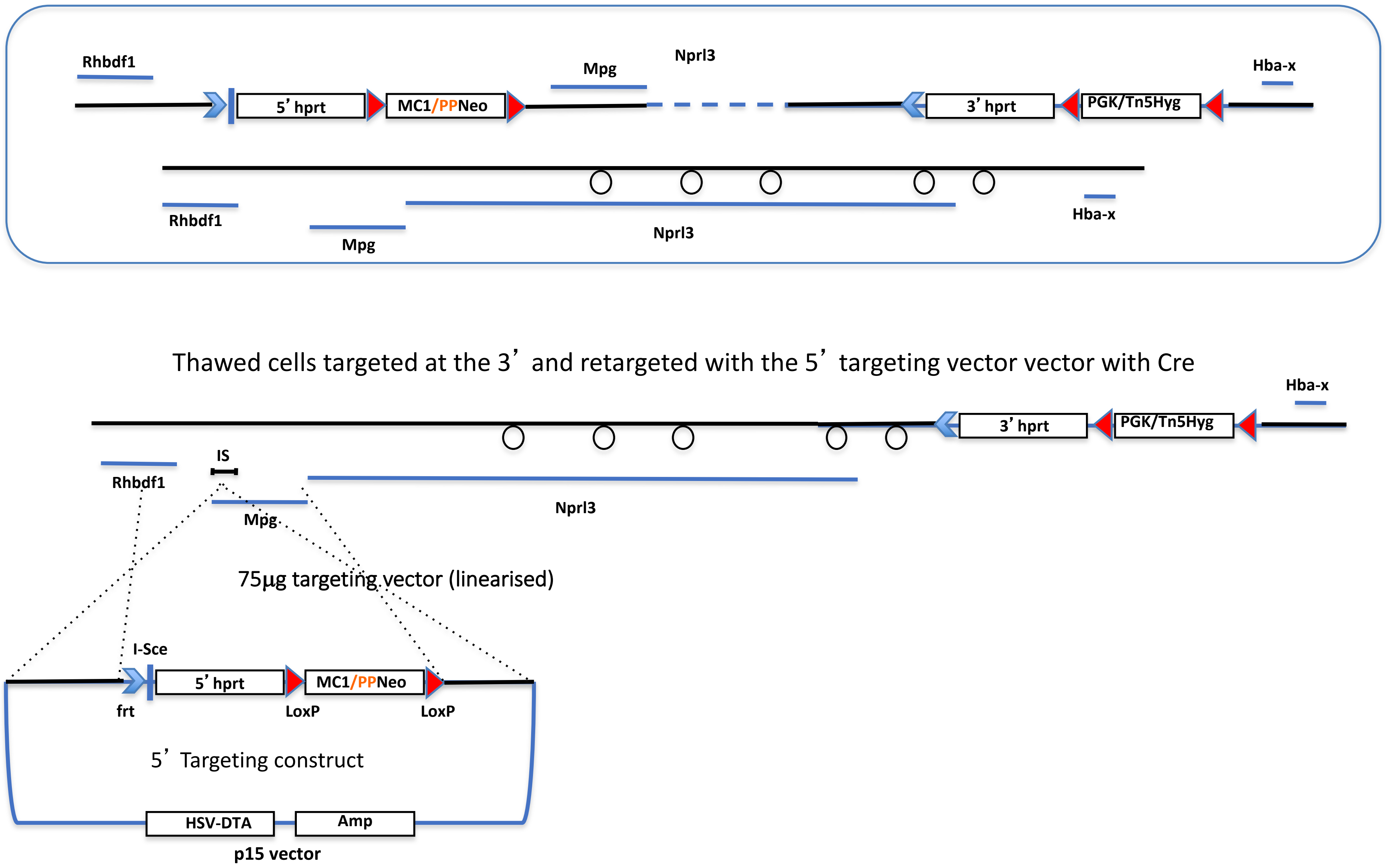

Thawed, expanded and frozen 24 positive clones of the 3' targeted clones

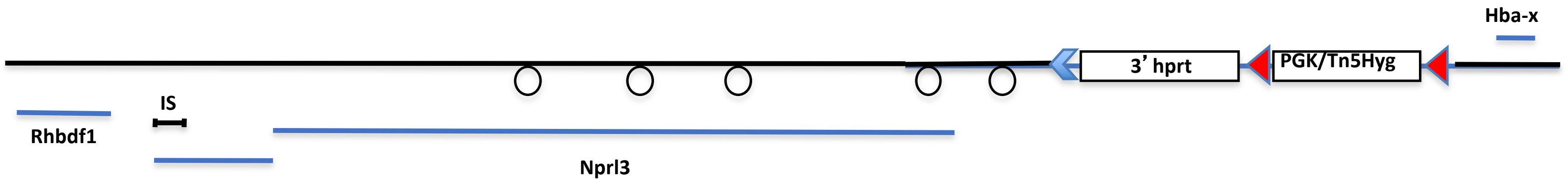

Clones 1, 2, 9, 10

- Expanded, frozen, DNA preps

- Karyotyped (20 spreads)

Cl1 75% with 40 Chr
Cl2 80%
Cl9 90%
Cl10 85%

- Transfected with T.V.#1 (5' end) + Cre

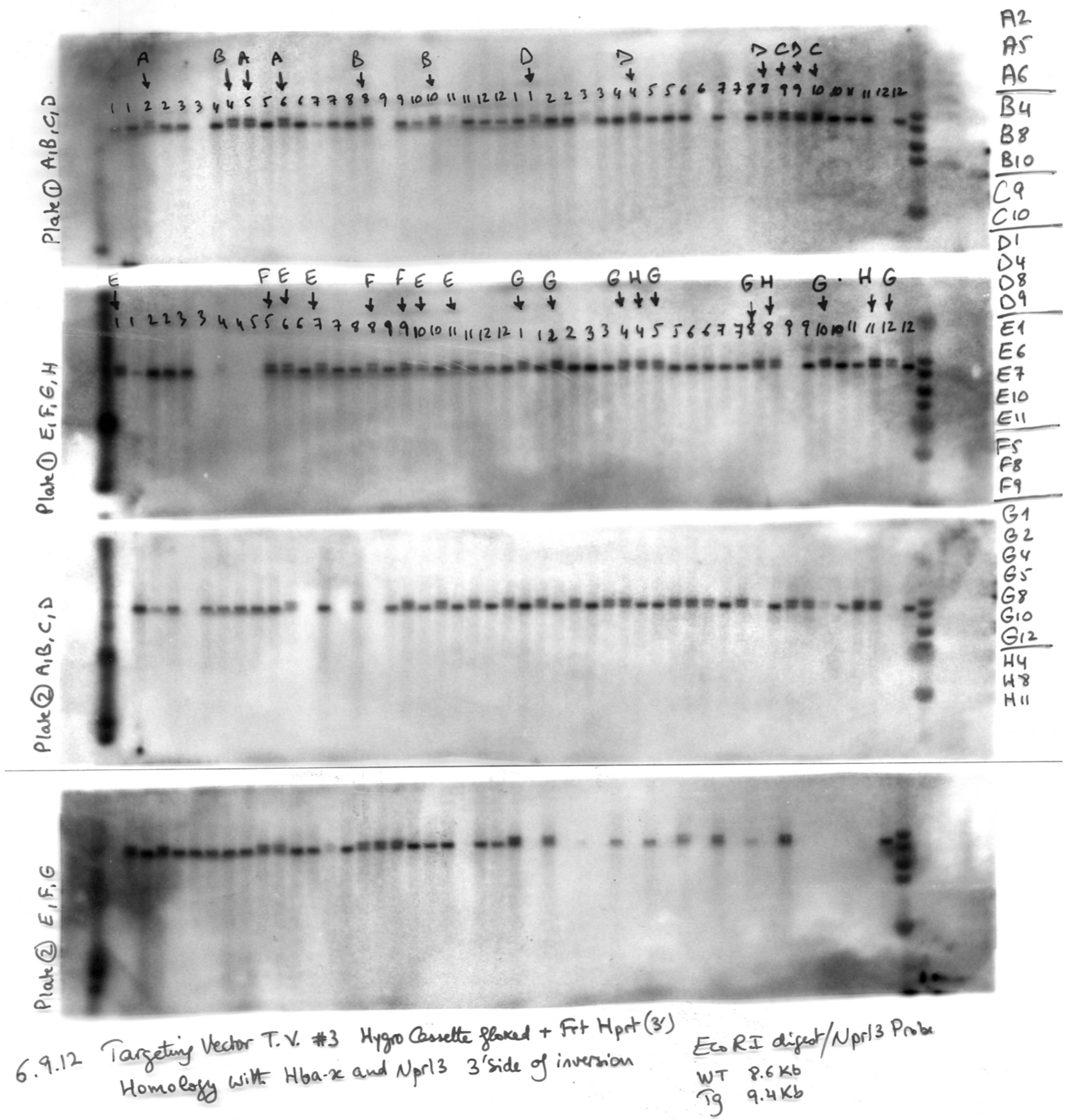

Double targeting in Cis

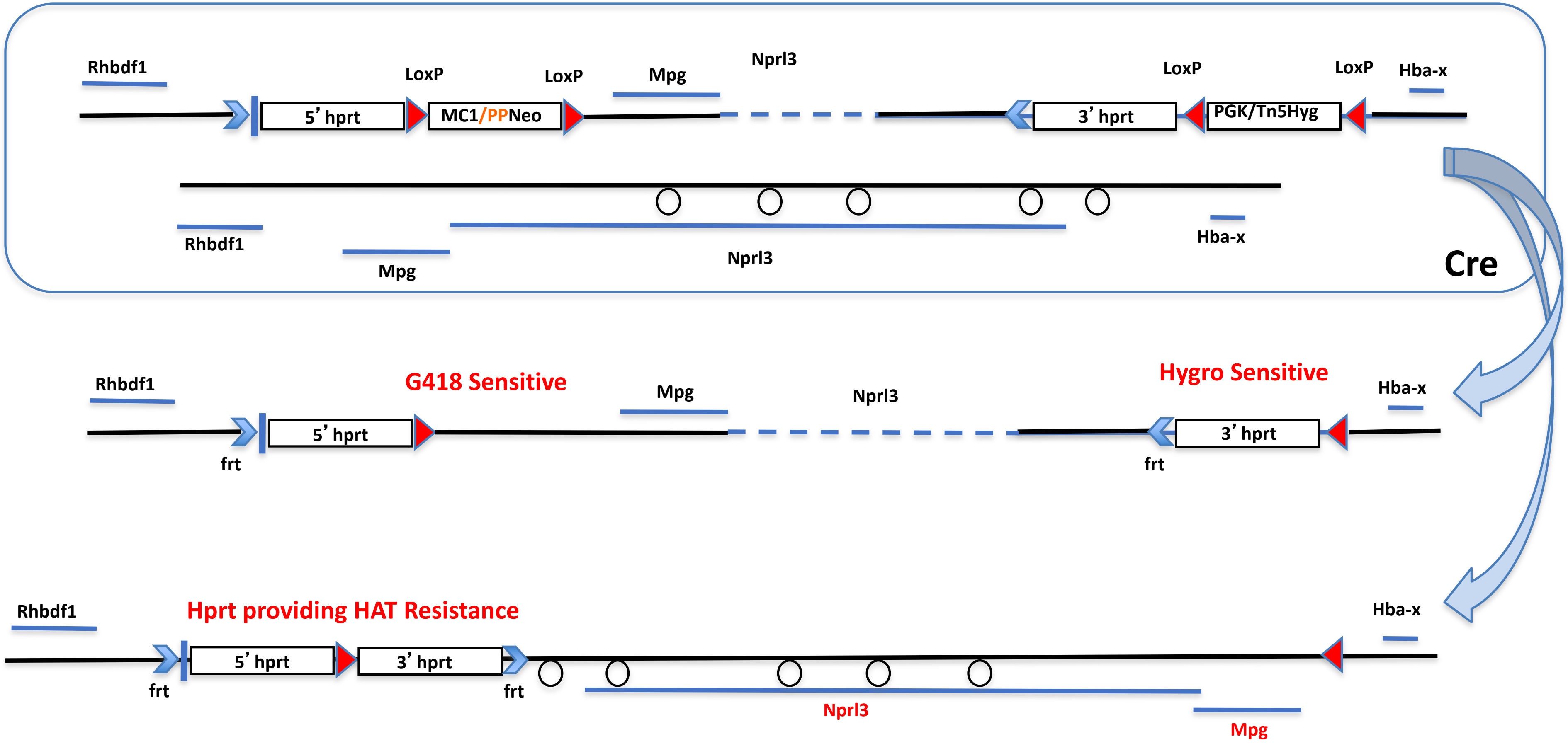

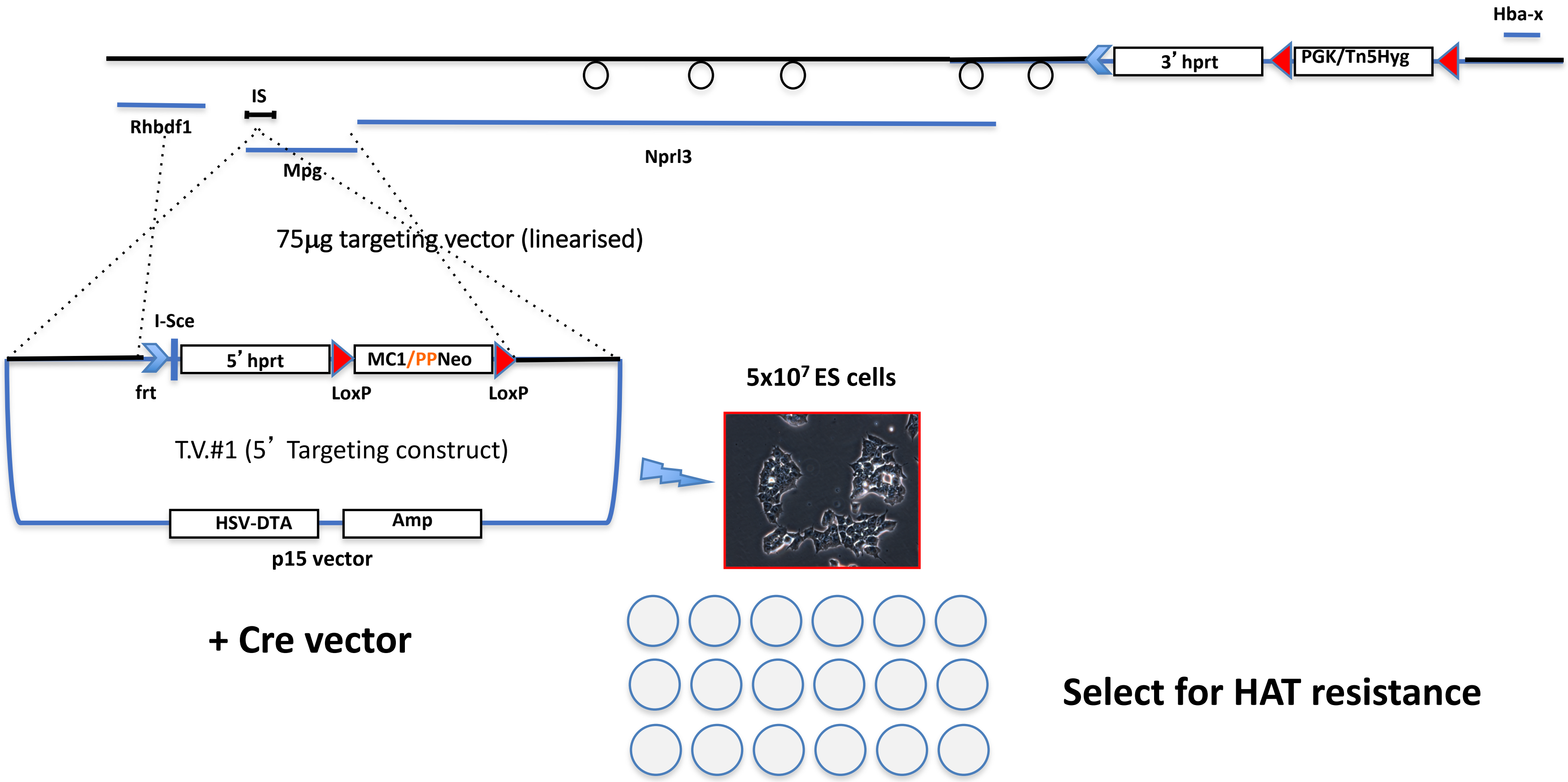

	Cl1	Cl2	Cl9	Cl10
	96-well	96-well	96-well	96-well
HAT Resistant	69	66	48	10
G418 Sensitive	67	66	48	10
Hygro Sensitive	67	66	45	9

screening by Southern Blot and PCR

	Cl1	Cl2	Cl9	Cl10
	96-well	96-well	96-well	96-well
HAT Resistant	69	66	48	10
G418 Sensitive	67	66	48	10
Hygro Sensitive	67	66	45	9

WT

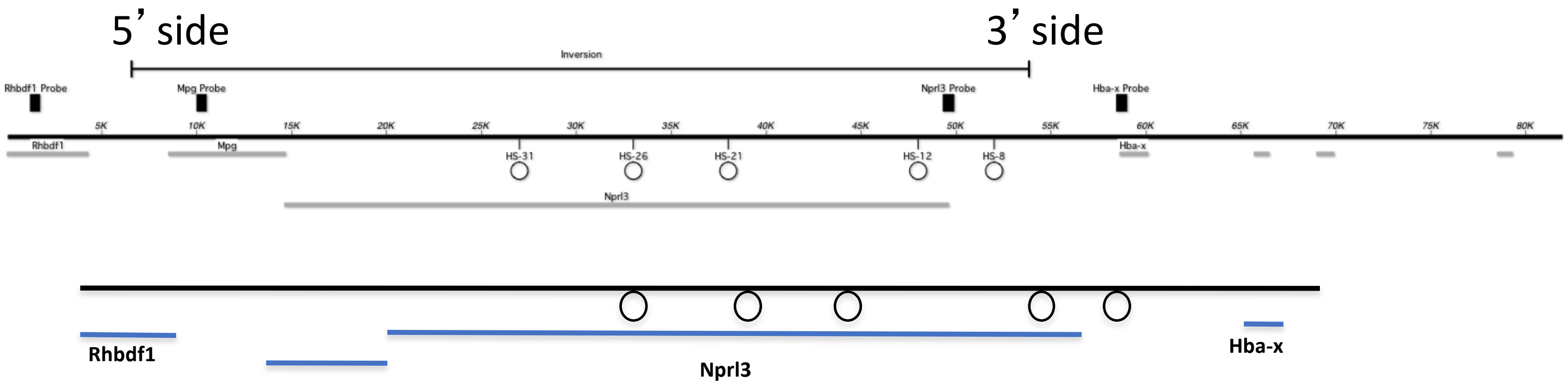

5' side
3' side
Inversion
Rhbdf1 Probe
Mpg Probe
Nprl3 Probe
Hba-x Probe
5K
10K
15K
20K
25K
30K
35K
40K
45K
50K
55K
60K
65K
70K
75K
80K
Rhbdf1
Mpg
Hba-x
HS-31
HS-26
HS-21
HS-12
HS-8
Nprl3
Rhbdf1
Nprl3
Hba-x

Inverted

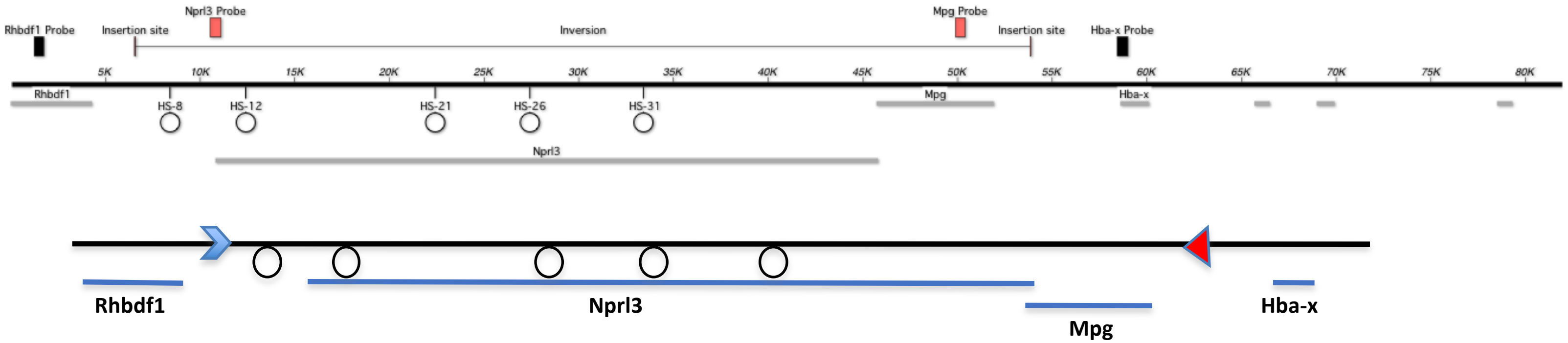

Nprl3 Probe
Mpg Probe
Rhbdf1 Probe
Insertion site
Inversion
Insertion site
Hba-x Probe
5K
10K
15K
20K
25K
30K
35K
40K
45K
50K
55K
60K
65K
70K
75K
80K
Rhbdf1
HS-8
HS-12
HS-21
HS-26
HS-31
Mpg
Hba-x
Nprl3
Rhbdf1
Nprl3
Mpg
Hba-x

Southern Blot
Screening strategies

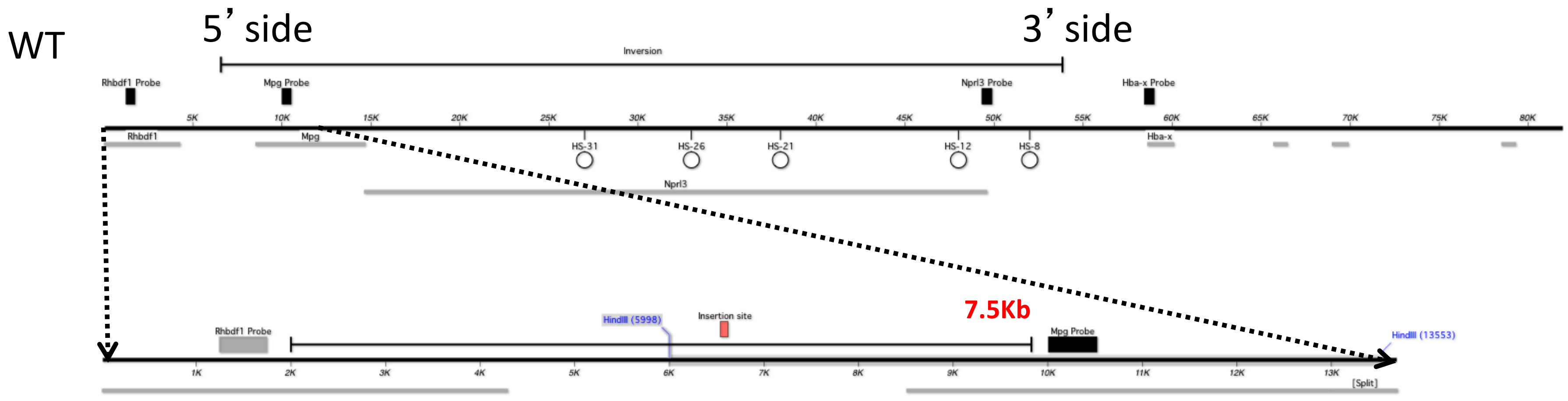

HindIII digest, Mpg probe

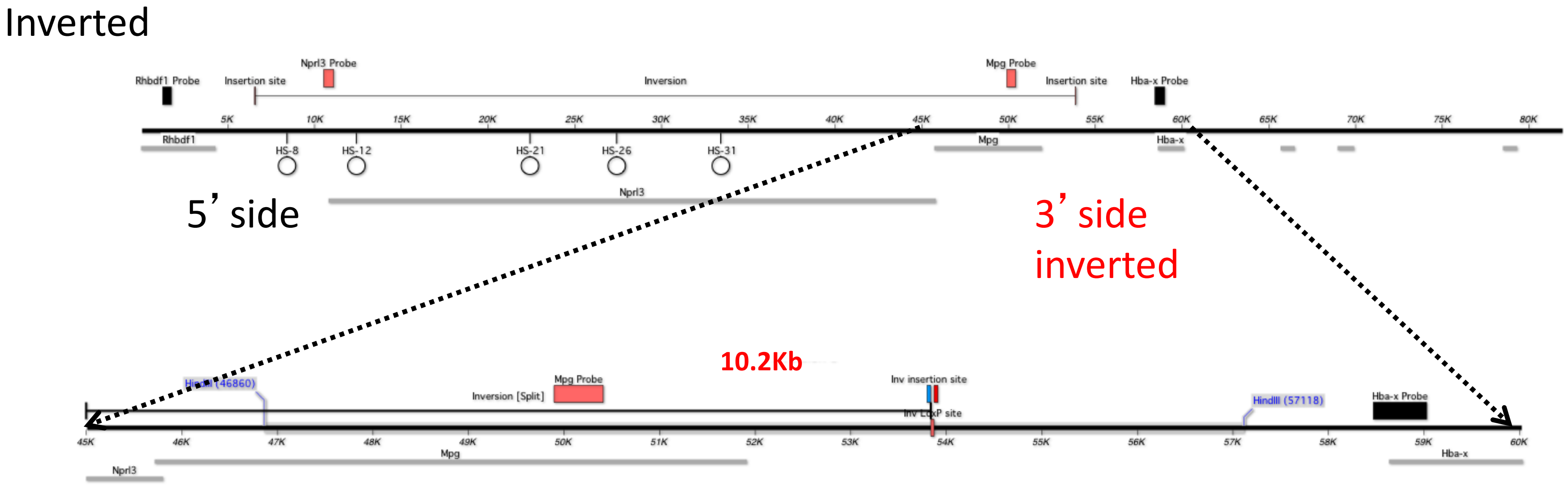

Examples of Screening the 5' Inverted site

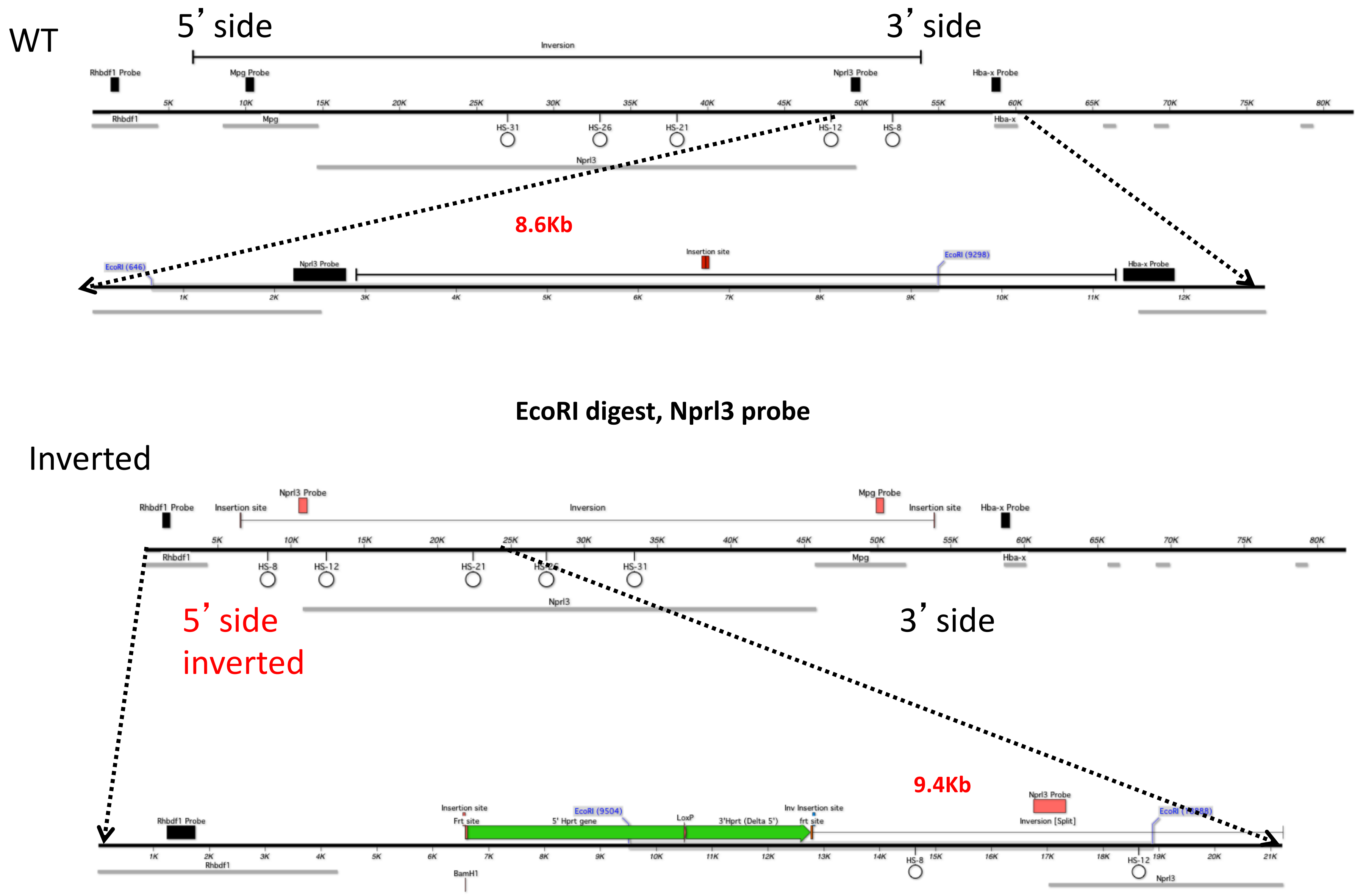

5’ side inverted as shown by Southern Blot

Cl1

Cl2

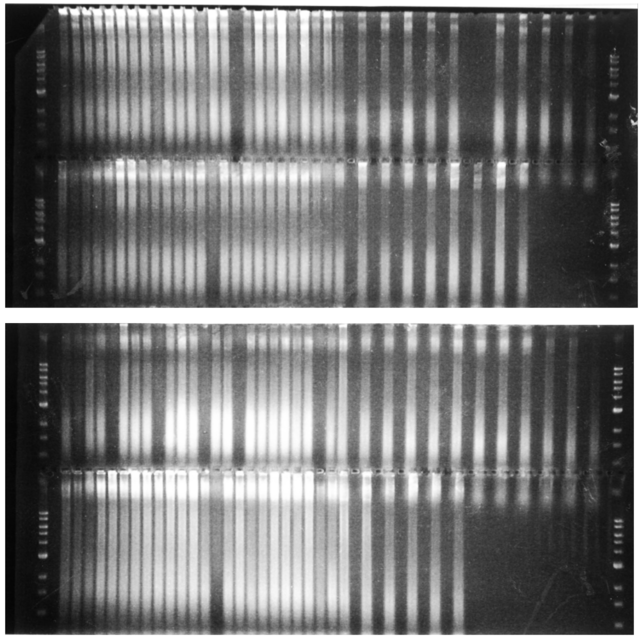

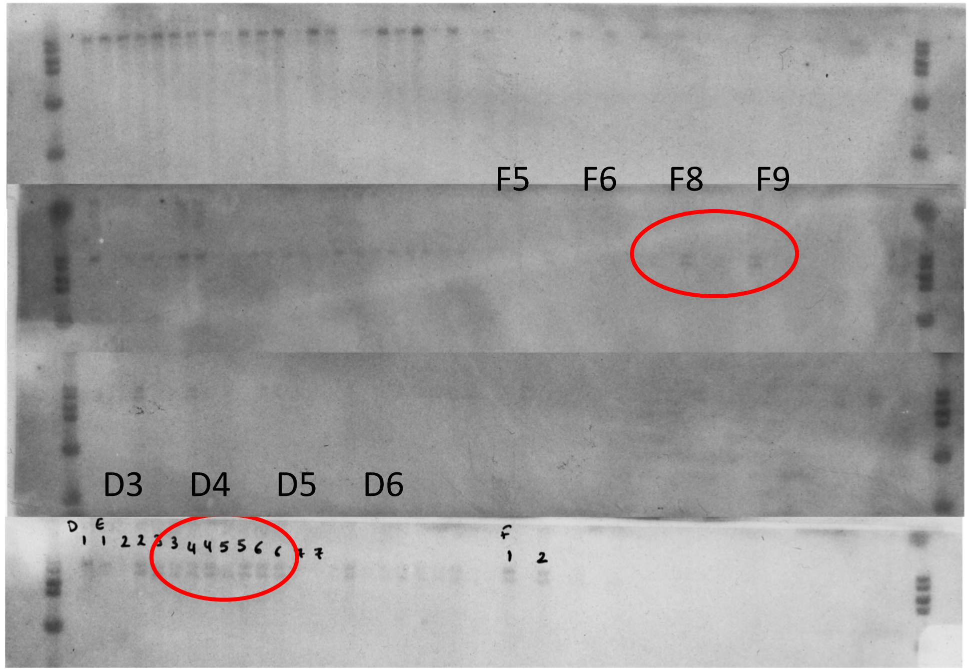

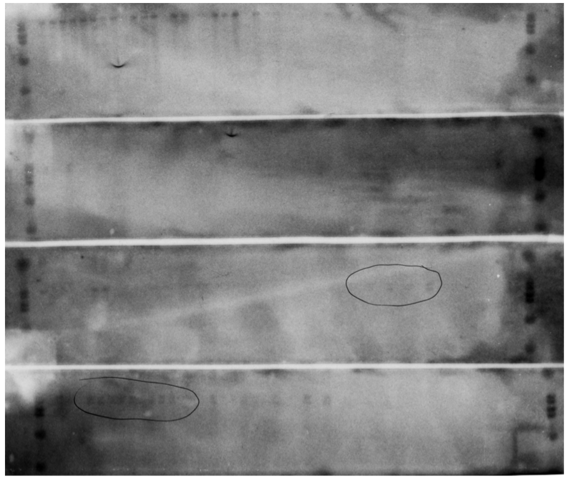

3' side inverted as shown by PCR

PCR strategy

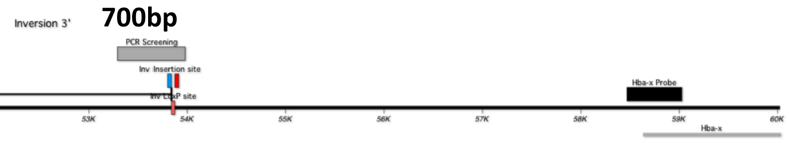

Cl1

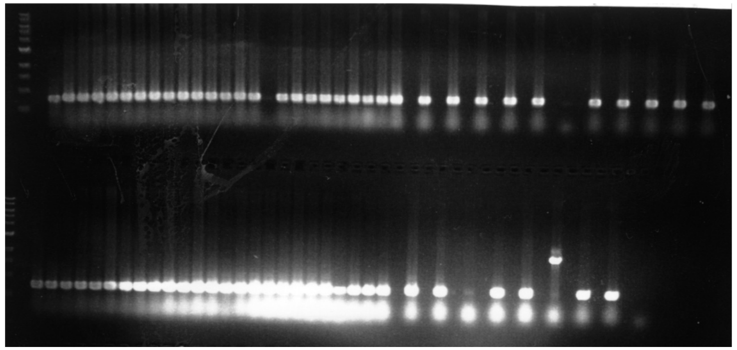

Cl2

F5 F6 F8 F9 D3 D4 D5 D6 WT Neg

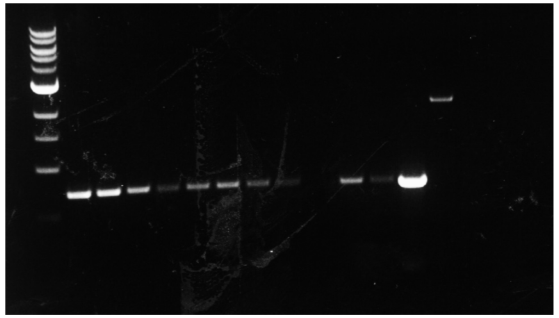

5’ side inverted

PCR strategy

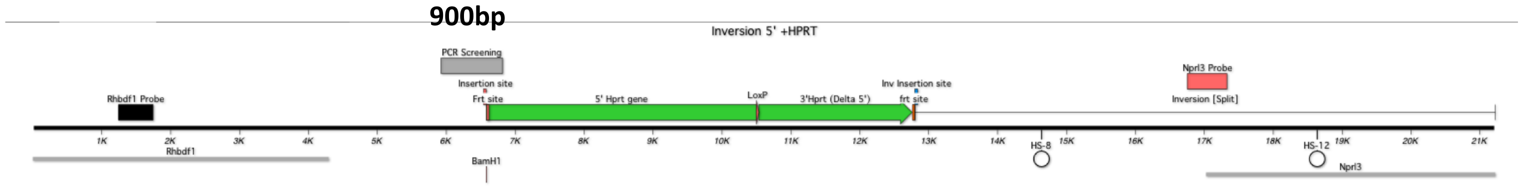

Cl1

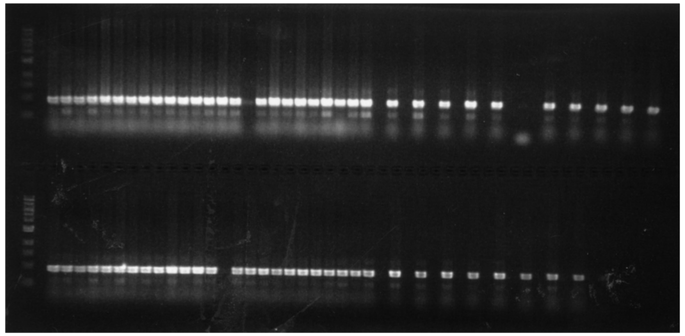

Cl2

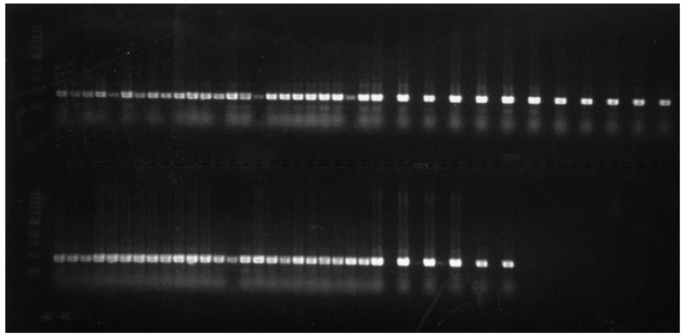

F5 F6 F8 F9 D3 D4 D5 D6 WT Neg

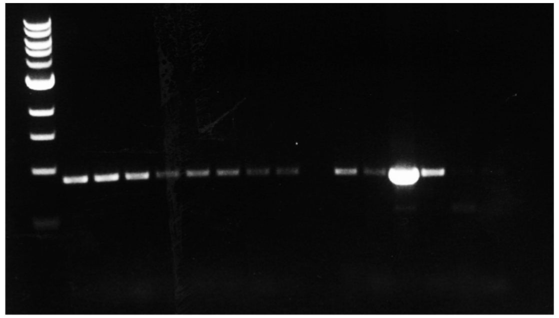

	Cl1	Cl2	Cl9	Cl10
	96-well	96-well	96-well	96-well
HAT Resistant	69	66	48	10
G418 Sensitive	67	66	48	10
Hygro Sensitive	67	66	45	9
PCR positive	69	66		
**Selected clones to expand and freeze**	F5 F6 F8 F9	D3 D4 D5 D6	A6 B3 C4 D5	H3 H4

transfect with FLP
Karyotyped

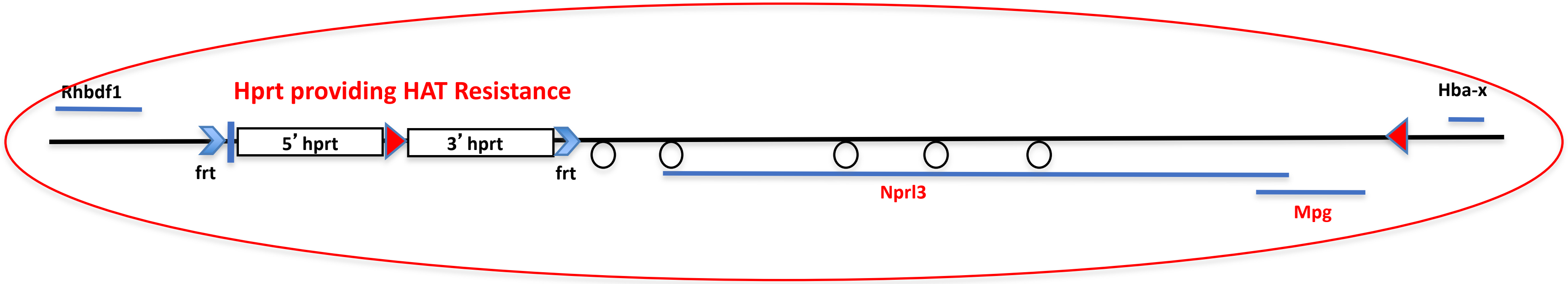

F6 and D3 selected for transfection with FLP vector

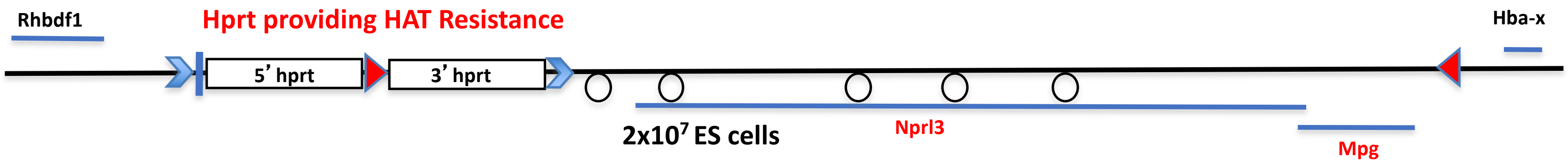

2x10^7 ES cells

50µg FLP vector

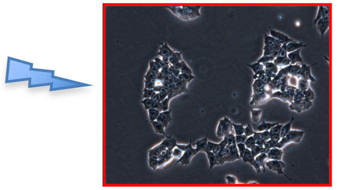

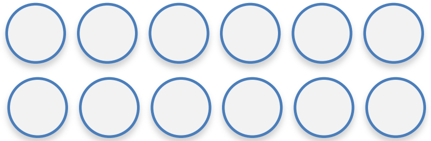

Plated cells at low concentration (10^4, 5x10^3, 2x10^3)
Added 6-TG 5 days after electroporation

Select for 6-TG resistance

Double targeting in Cis

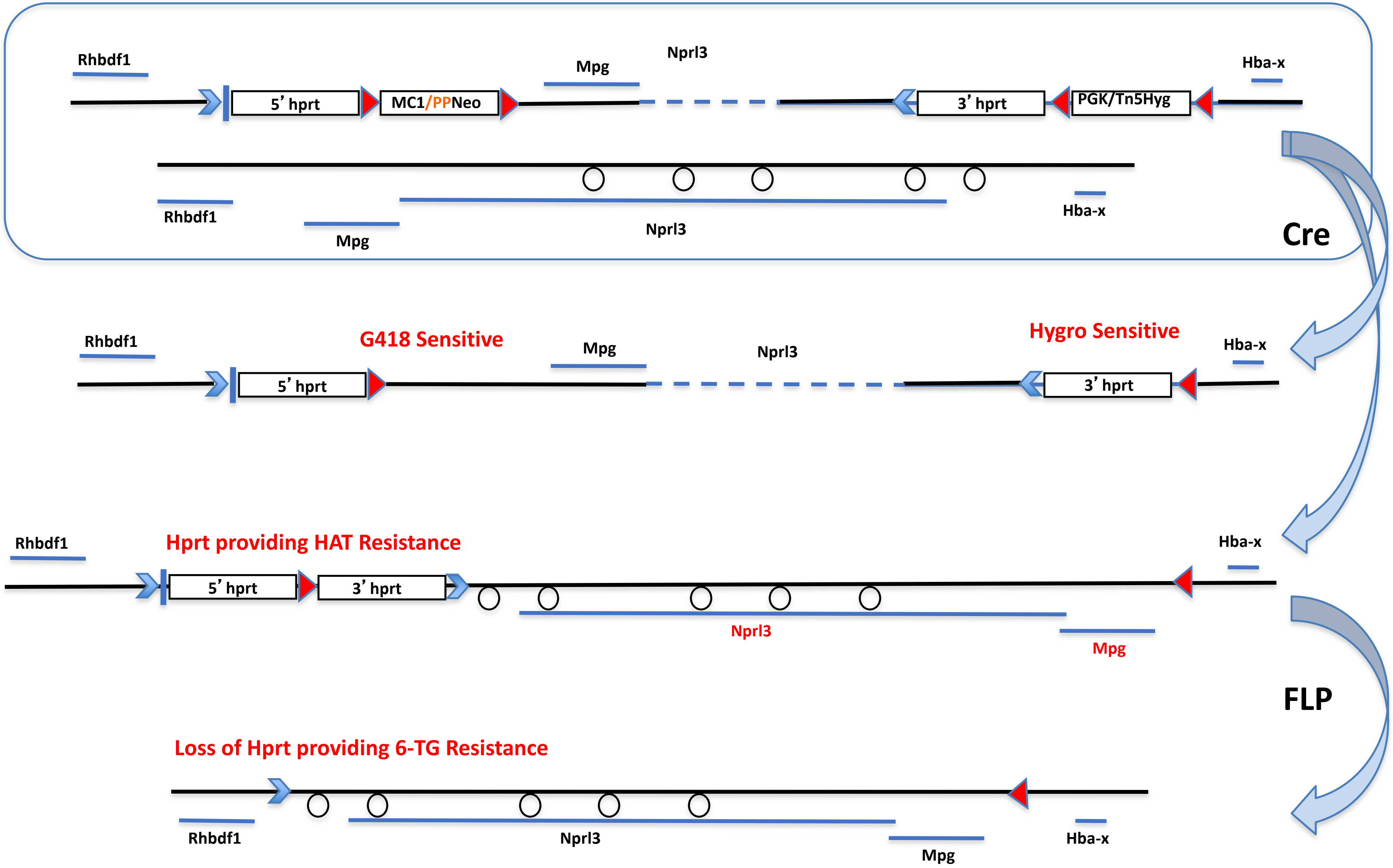

Data for the outcome of this screening is shown in Supplementary Fig. 12

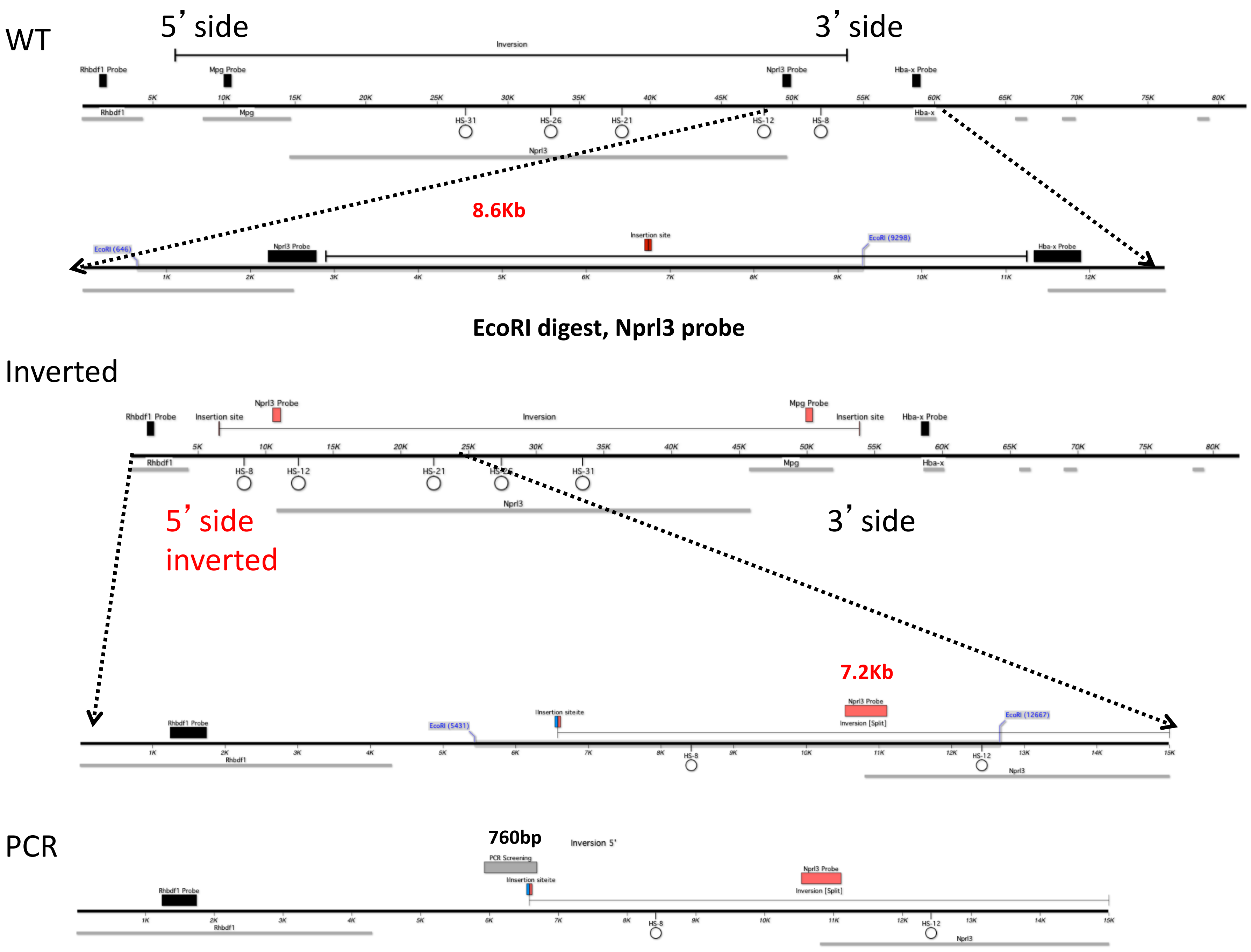

Summary of the clones produced and plan of action

Targeted at 3' side	Cl1	Cl2	Cl9	Cl10
	96-well	96-well	96-well	96-well
HAT Resistant	69	66	48	10
G418 Sensitive	67	66	48	10
Hygro Sensitive	67	66	45	9
PCR positive	69	66		
Southern Blot	?	?		
Inverted+HPRT	F6	D3	A6	H3

transfected with FLP (F6, D3)

Expanding (A6, H3)

Inverted-HPRT

For injection
