# Supplementary material for: The α-globin super-enhancer acts in an orientation-dependent manner
Source: Nat Commun. 2025 Jan 25;16:1033. doi: 10.1038/s41467-025-56380-1 (PMC11762767; doi:10.1038/s41467-025-56380-1)
Supplement: Supplementary file 5 — Reporting Summary [file 41467_2025_56380_MOESM5_ESM.pdf]

nature portfolio

Corresponding author(s): Doug Higgs and Mira Kassouf

Last updated by author(s): Dec 9, 2024

Reporting Summary

Nature Portfolio wishes to improve the reproducibility of the work that we publish. This form provides structure for consistency and transparency in reporting. For further information on Nature Portfolio policies, see our Editorial Policies and the Editorial Policy Checklist.

Statistics

For all statistical analyses, confirm that the following items are present in the figure legend, table legend, main text, or Methods section.

n/a	Confirmed	
[ ]	[x]	The exact sample size (*n*) for each experimental group/condition, given as a discrete number and unit of measurement
[ ]	[x]	A statement on whether measurements were taken from distinct samples or whether the same sample was measured repeatedly
[ ]	[x]	The statistical test(s) used AND whether they are one- or two-sided<br>*Only common tests should be described solely by name; describe more complex techniques in the Methods section.*
[x]	[ ]	A description of all covariates tested
[ ]	[x]	A description of any assumptions or corrections, such as tests of normality and adjustment for multiple comparisons
[ ]	[x]	A full description of the statistical parameters including central tendency (e.g. means) or other basic estimates (e.g. regression coefficient) AND variation (e.g. standard deviation) or associated estimates of uncertainty (e.g. confidence intervals)
[ ]	[x]	For null hypothesis testing, the test statistic (e.g. *F*, *t*, *r*) with confidence intervals, effect sizes, degrees of freedom and *P* value noted<br>*Give P values as exact values whenever suitable.*
[x]	[ ]	For Bayesian analysis, information on the choice of priors and Markov chain Monte Carlo settings
[x]	[ ]	For hierarchical and complex designs, identification of the appropriate level for tests and full reporting of outcomes
[x]	[ ]	Estimates of effect sizes (e.g. Cohen's *d*, Pearson's *r*), indicating how they were calculated

Our web collection on statistics for biologists contains articles on many of the points above.

Software and code

Policy information about availability of computer code

Data collection	Commercially available software was used for data collection:<br>- Blood parameters - Horiba Medical Scil Vet abc Plus+ instrument.<br>- Sequencing: Sanger Sequencing (ABI-3730 DNA analyser), Illumina Miseq, and NextSeq Platforms using the NextSeq System Suite (v2)<br>- FACS: Attune NxT software (v3.0)<br>- RT-qPCR: StepONEPlus Real-Time PCR system<br>- RNA (prior to any expression analysis) and DNA quality (prior to ATAC, ChIP, NG Capture C library preparations) assessment using TapeStation machine and software (Agilent).<br>- Bionano Genomics Saphyr system for long DNA optical imaging (cytogenetics); used to insure the INVEN genotype is normal apart from the engineered inversion.
Data analysis	All tools used and mentioned here are referenced in the text and in the Methods section.<br>- ChIP-seq and ATAC-seq data was processed using NGseqBasic (v20;https://www.biorxiv.org/content/10.1101/393413v1.article-info) which uses FASTQC (v0.11.4), UCSCtools (v3.8.5), and Samtools (v0.1.19) and also deepTools (v2.2.2).<br>- RNA-seq analysis:Reads were aligned to the mm9 mouse genome build using STAR. DeepTools bamCoverage was used to calculate normalized (RPKM) and strand-specific read coverage, which was visualized in the UCSC genome browser. Mapped RNA-seq reads were assigned to genes using Subread featureCounts using RefSeq gene annotation. Normalized differential gene expression, between biological triplicate data from littermate wild-type and INVEN mutant mice extracted in parallel, was calculated with the DESeq2 R package.<br>- Chromosome Conformation Capture data was processed using CaptureCompendium which includes CCseqBasicS (https://github.com/Hughes-Genome-Group/CCseqBasicS) and CaptureCompare (https://github.com/Hughes-Genome-Group/CaptureCompare).<br>- FACS was analysed in FlowJo (v10.7).

- Modified sequences in genetically engineered mESCs were checked using Sasquatch tools for undesirable creation of potentially active hypersensitive sites in erythroid cells (Schwessinger, R. et al. DeepC: predicting 3D genome folding using megabase-scale transfer learning. Nat Methods 17, 1118–1124 (2020)).
- Bionano Genomics Saphyr system software to analyse Bionano data.
- Statistical analysis using unpaired t-test or one-way or two-way ANOVA with Tukey post-hoc or Sidak multiple comparisons tests were used as specified in figure legends.

For manuscripts utilizing custom algorithms or software that are central to the research but not yet described in published literature, software must be made available to editors and reviewers. We strongly encourage code deposition in a community repository (e.g. GitHub). See the Nature Portfolio guidelines for submitting code & software for further information.

Data

Policy information about availability of data

All manuscripts must include a data availability statement. This statement should provide the following information, where applicable:

- Accession codes, unique identifiers, or web links for publicly available datasets
- A description of any restrictions on data availability
- For clinical datasets or third party data, please ensure that the statement adheres to our policy

All data generated for this study are included in this published article and its supplementary information. Source data are provided as a Source Data file with this paper. ChIP-seq, ATAC-seq, RNA-seq and NG Capture-C data (sequence reads and processed files) is available in the Gene Expression Omnibus (GEO) under accession numbers GSE184435 (https://www.ncbi.nlm.nih.gov/geo/query/acc.cgi?acc=GSE184435) and GSE211238 (https://www.ncbi.nlm.nih.gov/geo/query/acc.cgi?acc=GSE211238). In addition to raw and processed files accessible through GEO, NG Capture C data and visualization for the 42 promoter captures are available on this link:
https://capturesee.molbiol.ox.ac.uk/projects/capture_compare/3950.

Human research participants

Policy information about studies involving human research participants and Sex and Gender in Research.

Reporting on sex and gender	*Use the terms sex (biological attribute) and gender (shaped by social and cultural circumstances) carefully in order to avoid confusing both terms. Indicate if findings apply to only one sex or gender; describe whether sex and gender were considered in study design whether sex and/or gender was determined based on self-reporting or assigned and methods used. Provide in the source data disaggregated sex and gender data where this information has been collected, and consent has been obtained for sharing of individual-level data; provide overall numbers in this Reporting Summary. Please state if this information has not been collected. Report sex- and gender-based analyses where performed, justify reasons for lack of sex- and gender-based analysis.*
Population characteristics	*Describe the covariate-relevant population characteristics of the human research participants (e.g. age, genotypic information, past and current diagnosis and treatment categories). If you filled out the behavioural & social sciences study design questions and have nothing to add here, write "See above."*
Recruitment	*Describe how participants were recruited. Outline any potential self-selection bias or other biases that may be present and how these are likely to impact results.*
Ethics oversight	*Identify the organization(s) that approved the study protocol.*

Note that full information on the approval of the study protocol must also be provided in the manuscript.

Field-specific reporting

Please select the one below that is the best fit for your research. If you are not sure, read the appropriate sections before making your selection.

[x] Life sciences [ ] Behavioural & social sciences [ ] Ecological, evolutionary & environmental sciences

For a reference copy of the document with all sections, see nature.com/documents/nr-reporting-summary-flat.pdf

Life sciences study design

All studies must disclose on these points even when the disclosure is negative.

Sample size	- For blood parameters, at least 6 mice were analysed per genotype to allow statistical testing using one-way ANOVA with Tukey post-hoc test. Sample sizes were chosen with a view to detecting 25% reduction in hemoglobin concentration.<br>- For ChIP-seq, ATAC-seq, RNA-seq and NG Capture-C data, three independent replicates of each genotype were analysed to enable statistical analysis with DESeq2.<br>- For RNA expression using RT-PCR, at least three independent replicates of each genotype were analysed to allow statistical analysis (ANOVA).
Data exclusions	No data was excluded.

Replication	Data from primary cells derived from adult mouse tissues were reproducible in each genotype (blood parameters, spleen-derived data). Data from engineered mouse Embryonic Stem Cell (ESC) lines were reproducible across experimental and biological replicas. Two independent INVEN cell line were originally generated and analysed (A4.2 and B4.4 or F11-2) to eliminate clonal variation and confirm that the phenotype is caused specifically by the engineered inversion. These produced identical phenotypes. The subsequently derived genetic models were assessed similarly (independent clones when possible as well as experimental replicas).
Randomization	Randomisation was not applicable to this study as comparison was between distinct genotypes.
Blinding	Blinding was only applied to adult mice blood assessment; scoring of BCB-stained smears for retic count and analysis of blood parameters using the blood analyzer. Counts were made by two independent assessors blinded to genotype.

Reporting for specific materials, systems and methods

We require information from authors about some types of materials, experimental systems and methods used in many studies. Here, indicate whether each material, system or method listed is relevant to your study. If you are not sure if a list item applies to your research, read the appropriate section before selecting a response.

Materials & experimental systems

n/a	Involved in the study
☐	☒ Antibodies
☐	☒ Eukaryotic cell lines
☒	☐ Palaeontology and archaeology
☐	☒ Animals and other organisms
☒	☐ Clinical data
☒	☐ Dual use research of concern

Methods

n/a	Involved in the study
☐	☒ ChIP-seq
☐	☒ Flow cytometry
☒	☐ MRI-based neuroimaging

Antibodies

Antibodies used	ChIP:<br>H3K27ac (Abcam ab4729, 0.5 µg/ml)<br>H3K4me1 (Abcam ab8895, 0.3 µg/ml)<br>H3K4me3 (Abcam 8580, 2.5 µg/ml)<br>H3K27me3 (Cell Signaling 9733, 2 µg/ml)<br>CTCF Merck (Millipore 07-729, 5 µg/ml)<br>Rad21 Abcam (ab154769, 5 µg/ml)<br>Anti-Med1 (Bethyl A300-793A (Lot 11), 5 µg/ml)<br>Anti-Rpd1-NTD (Cell signalling 14958 (D8L4Y) 5 µg/ ~250,000 cells)<br><br>Flow cytometry:<br>FITC Rat Anti-Mouse CD71 (eBioscience 11-0711-85 2.5 µg/ml)<br>PE Rat Anti-Mouse Ter119 (BD Pharmingen 553673 2 µg/ml)<br>Hoechst (Invitrogen H3569 1 µg/ml)
Validation	Data generated from all antibodies used in this study have been validated in various ways: at the basic level; by the manufacturers, and more thoroughly on our specific cell types as data has been produced (reproduced) in our laboratory by other scientists for other projects and by published literature by us and others on similar cell types. In some instances, the differences in phenotypes based on the genotype confirm specificity; when deleting CTCF sites or a gene promoter for example and these deletions result in absence of the relevant ChIP peak (CTCF ChIP, H3K4me3) and when a gene is reactivated it resulted in H3K27me3 disappearing and H3K4me3 appearing. in the inversion models, Rad21, Med1 and PolII binding pattern reflects the gene expression profiles.

Eukaryotic cell lines

Policy information about cell lines and Sex and Gender in Research

Cell line source(s)	The wildtype E14TG2a mouse Embryonic Stem Cell (mESC) line was a kind gift from Prof Andrew Smith (also co-author).
Authentication	In addition to the conventional tests (mESC colony morphology and alkaline phosphatase staining), the pluripotency of mESCs was verified in the in vitro hematopoietic differentiation assay which only produces embryoid bodies harbouring the three germ layers including the mesoderm that then produces erythroid cells if they were pluripotent. All the mESCs used passed this test. The SEINV mESCs generated a mouse model, the ultimate test of pluripotency.<br>The Genetic engineering outcome was assessed using various methods: original INVEN clones were assessed following a thorough Southern blot strategy. Genome integrity of the SEINV mESCs was confirmed using the Bionano Saphyr system optical imaging, a cytogenomics tool that captures long DNA fragments across the genomes and detect structural variants at

	high sensitivity and resolution. Other methods include PCR genotyping, restriction digests on PCR products, Sanger sequencing, and various genomics assays confirm the engineered modifications (ATAC-seq, ChIP-seq, RNA-seq, Capture-C).
Mycoplasma contamination	Mycoplasma testing is regularly done within the tissue culture facility where these lines were produced, handled and analysed (MRC-MHU Weatherall Institute of Molecular Medicine)
Commonly misidentified lines (See ICLAC register)	No commonly misidentified lines were used.

Animals and other research organisms

Policy information about studies involving animals; ARRIVE guidelines recommended for reporting animal research, and Sex and Gender in Research

Laboratory animals	C57BL/6J mice were sourced from MRC Harwell/Charles River Laboratories. All mouse work was performed in accordance with UK Home office regulations, under the appropriate animal licenses. Mouse model generation and animal husbandry was conducted by the Mouse Transgenics Core Facility at the Weatherall Institute of Molecular Medicine.
Wild animals	No wild animals were used in this study.
Reporting on sex	Sex was not relevant to this study however when adult mice were used we used both males and females.
Field-collected samples	We had no field-collected samples.
Ethics oversight	No ethical approvals were needed for this study. We abide by the Home Office Regulations for the mouse work under the appropriate license.

Note that full information on the approval of the study protocol must also be provided in the manuscript.

ChIP-seq

Data deposition

- [x] Confirm that both raw and final processed data have been deposited in a public database such as GEO.
- [ ] Confirm that you have deposited or provided access to graph files (e.g. BED files) for the called peaks.

Data access links *May remain private before publication.*	Raw and processed files are available on the Gene Expression Omnibus (GEO) accession number: GSE211238
Files in database submission	15EBctcfA_R1.fastq.gz<br>16EBctcfB_R1.fastq.gz<br>22EBA3CTCF_R1.fastq.gz<br>23EBA8CTCF_R1.fastq.gz<br>25EBC2CTCF_R1.fastq.gz<br>27EBC12CTCF_R1.fastq.gz<br>20CTCFChIP151_R1.fastq.gz<br>21CTCFChIP152_R1.fastq.gz<br>1InvenH3K4me3A_R1.fastq.gz<br>2InvenH3K4me3B_R1.fastq.gz<br>10InvenH3K4meC_R1.fastq.gz<br>4InvenH3K27me3A_R1.fastq.gz<br>8InvenH3K27me3B_R1.fastq.gz<br>9InvenH3K27me3C_R1.fastq.gz<br>15EBctcfA_R2.fastq.gz<br>16EBctcfB_R2.fastq.gz<br>22EBA3CTCF_R2.fastq.gz<br>23EBA8CTCF_R2.fastq.gz<br>25EBC2CTCF_R2.fastq.gz<br>27EBC12CTCF_R2.fastq.gz<br>20CTCFChIP151_R2.fastq.gz<br>21CTCFChIP152_R2.fastq.gz<br>1InvenH3K4me3A_R2.fastq.gz<br>2InvenH3K4me3B_R2.fastq.gz<br>10InvenH3K4meC_R2.fastq.gz<br>4InvenH3K27me3A_R2.fastq.gz<br>8InvenH3K27me3B_R2.fastq.gz<br>9InvenH3K27me3C_R2.fastq.gz<br>CD71_EB_Rad21_INVEN_dCTCF_dMpg_Rep1_R1.fastq.gz<br>CD71_EB_Rad21_INVEN_dCTCF_dMpg_Rep1_R2.fastq.gz<br>CD71_EB_Rad21_INVEN_dCTCF_dMpg_Rep2_R1.fastq.gz

CD71_EB_Rad21_INVEN_dCTCF_dMpg_Rep2_R2.fastq.gz
CD71_EB_Rad21_INVEN_Rep1_R1.fastq.gz
CD71_EB_Rad21_INVEN_Rep1_R2.fastq.gz
CD71_EB_Rad21_INVEN_Rep2_R1.fastq.gz
CD71_EB_Rad21_INVEN_Rep2_R2.fastq.gz
CD71_EB_Rad21_WT_E14_Rep1_R1.fastq.gz
CD71_EB_Rad21_WT_E14_Rep1_R2.fastq.gz
CD71_EB_Rad21_WT_E14_Rep2_R1.fastq.gz
CD71_EB_Rad21_WT_E14_Rep2_R2.fastq.gz
CD71_EB_B12_INVEN_dCTCF_dMpg_Brd4_ChIPment_R1.fastq.gz
CD71_EB_B12_INVEN_dCTCF_dMpg_Brd4_ChIPment_R2.fastq.gz
CD71_EB_B12_INVEN_dCTCF_dMpg_PolII_ChIPment_R1.fastq.gz
CD71_EB_B12_INVEN_dCTCF_dMpg_PolII_ChIPment_R2.fastq.gz
CD71_EB_E14_WT_PolII_ChIPment_R1.fastq.gz
CD71_EB_E14_WT_PolII_ChIPment_R2.fastq.gz
EB_CD71_INVEN_CTCF_1.bw
EB_CD71_CTCF_KO_CTCF_1.bw
EB_CD71_CTCF_KO_CTCF_2.bw
EB_CD71_INVEN_KO_CTCF_1.bw
EB_CD71_INVEN_KO_CTCF_2.bw
APHSpleen_INVEN_Ter119_CTCF_1.bw
APHSpleen_INVEN_Ter119_CTCF_2.bw
APHSpleen_INVEN_Ter119_H3K4me3_1.bw
APHSpleen_INVEN_Ter119_H3K4me3_2.bw
APHSpleen_INVEN_Ter119_H3K4me3_3.bw
APHSpleen_INVEN_Ter119_H3K27me3_1.bw
APHSpleen_INVEN_Ter119_H3K27me3_2.bw
APHSpleen_INVEN_Ter119_H3K27me3_3.bw
CD71_EB_Rad21_INVEN_dCTCF_dMpg_Rep1.rpkm.bw
CD71_EB_Rad21_INVEN_dCTCF_dMpg_Rep2.rpkm.bw
CD71_EB_Rad21_INVEN_Rep1.rpkm.bw
CD71_EB_Rad21_INVEN_Rep2.rpkm.bw
CD71_EB_Rad21_WT_E14_Rep1.rpkm.bw
CD71_EB_Rad21_WT_E14_Rep2.rpkm.bw
CD71_EB_B12_INVEN_dCTCF_dMpg_Brd4_ChIPment.rpkm.bw
CD71_EB_B12_INVEN_dCTCF_dMpg_PolII_ChIPment.rpkm.bw
CD71_EB_E14_WT_PolII_ChIPment.rpkm.bw

Genome browser session
(e.g. UCSC)

Methodology

Replicates

At least two replicates were performed per ChIP as indicated in the text.

Sequencing depth

chip_inven_spleen_h3k4me3_1: 77312564 total reads; 65088964 mapped reads
chip_inven_spleen_h3k4me3_2: #84579480 total; 68005816 mapped
chip_inven_spleen_h3k4me3_3: #67758664 total; 52832808 mapped
chip_inven_spleen_h3k27me3_1: #75368622 total; 58496302 mapped
chip_inven_spleen_h3k27me3_2: #76041504 total; 60852918 mapped
chip_inven_spleen_h3k27me3_3: #67370460 total; 51310698 mapped
chip_inven_spleen_ctcf_1: #102408418 total; 74896168 mapped
chip_inven_spleen_ctcf_2: #108388964 total; 80683144 mapped
chip_inven_ctcf_dko_ebd7_ctcf_C2: #74593058 total; 54293158 mapped
chip_inven_ctcf_dko_ebd7_ctcf_C12: #77224348 total; 57827716 mapped
CD71_EB_Rad21_INVEN_dCTCF_dMpg_Rep1: #49118754 total; 47360302 mapped
CD71_EB_Rad21_INVEN_dCTCF_dMpg_Rep2: # 37173267 total 35563664 mapped
CD71_EB_Rad21_INVEN_Rep1: #55377971 total 53478506 mapped
CD71_EB_Rad21_INVEN_Rep2: #47750316 total 45940579 mapped
CD71_EB_Rad21_Mira_INVEN_A4.2_Rep1: #55377971 total 53722169 mapped
CD71_EB_Rad21_Mira_INVEN_A4.2_Rep2: #47750316 total 46126805 mapped
CD71_EB_Rad21_Mira_INVEN_B12_Rep1: #49118754 total 47625543 mapped
CD71_EB_Rad21_Mira_INVEN_B12_Rep2: #37173267 total 35704922 mapped
CD71_EB_Rad21_Mira_INVEN_E14_Rep1: #36511235 total 34408187 mapped
CD71_EB_Rad21_Mira_INVEN_E14_Rep2: #39166447 total 37697705 mapped
CD71_EB_Rad21_WT_E14_Rep1: #36511235 total 34229282 mapped
CD71_EB_Rad21_WT_E14_Rep2: #39166447 total 37529289 mapped
CD71_EB_B12_INVEN_dCTCF_dMpg_Brd4_ChIPment: #47511758 total 46789579 mapped
CD71_EB_B12_INVEN_dCTCF_dMpg_PolII_ChIPment: #43528607 total 42475214 mapped

	CD71_EB_E14_WT_PolII_ChIPment: #42753309 total 41663099 mapped
Antibodies	Antibodies and concentrations used as specified in the antibody field above.
Peak calling parameters	Bigwig coverage tracks for Rad21, Med1 and PolII ChIP were used as inputs for LanceOtron12 callpeaks using default settings. The resulting peak regions were filtered for peaks with scores greater than 0.8 and concatenated across samples using bedtools intersect and bedtools merge functions to create a shared peak list with no repeated regions.
Data quality	ChIP quality was assessed in two ways; ChIP material was tested by qPCR prior to sequencing to ensure a good fold-enrichment at a relevant (expected positive) sequence over a negative region. Replicates were also checked for consistency. For the erythroid populations, we also checked that WT and engineered cells tested were yielding the expected ChIP profile over well-characterised unaffected regions (the beta globin and mitoferrin loci).
Software	Sequencing data was collected using MiSeq or NextSeq Illumina platforms and mapped using bowtie2.

Flow Cytometry

Plots

Confirm that:

☒ The axis labels state the marker and fluorochrome used (e.g. CD4-FITC).

☒ The axis scales are clearly visible. Include numbers along axes only for bottom left plot of group (a 'group' is an analysis of identical markers).

☒ All plots are contour plots with outliers or pseudocolor plots.

☒ A numerical value for number of cells or percentage (with statistics) is provided.

Methodology

Sample preparation	Minimum of 10^5 cells were collected per sample. Cells will spun down and resuspended in PBS/10%FCS (200 µl) and antibodies added at concentrations set above in antibody section, incubated at 4 degrees C for 20 minutes then washed once and resuspended in 500 µl of PBS/10%FCS with Hoechst.
Instrument	Attune NxT Flow Cytometer
Software	Data collection: Attune NxT software (v3.0)<br>Data analysis: FlowJo v10.7
Cell population abundance	Analysis of erythroid cell populations was done based on CD71/ter119 conventional analysis with the expected population frequency from the various cell populations analysed (consitent with published data by us and others, referenced in the text).
Gating strategy	Voltages and compensations were set using unstained and single fluorophore-stained samples. Negative and positive populations were established following the software's criteria and the following plots: Forward and Side Scatter (to gate the live and right size populations), doublet exclusion was done by gating single cells using FCS-area and FCS-height, and live cells were selected using the Hoechst negative gate. The main fluorophore compensation was done between Ter119-PE and CD71-FITC.

☒ Tick this box to confirm that a figure exemplifying the gating strategy is provided in the Supplementary Information.
